# High-Performance Cu-Based Liquid Thermocells Enabled by Thermosensitive Crystallization and Etched Carbon Cloth Electrode

**DOI:** 10.1007/s40820-025-01977-w

**Published:** 2026-01-05

**Authors:** Wei Fang, Zeping Ou, Yifan Wang, Zhe Li, Qian Huang, Pengchi Zhang, Xinzhe Li, Yujie Zheng, Lijun Hu, Chen Li, Jianyong Ouyang, Kuan Sun

**Affiliations:** 1 MOE Key Laboratory of Low-Grade Energy Utilization Technologies and Systems, School of Energy and Power Engineering, Chongqing University, Chongqing, 400044 People’s Republic of China; 2 Hunan Key Laboratory for the Design and Application of Actinide Complexes, School of Chemistry and Chemical Engineering, University of South China, Hengyang, 421001 Hunan People’s Republic of China; 3 Sibley School of Mechanical and Aerospace Engineering, Cornell University, Ithaca, NY 14853 USA; 4 Department of Materials Science and Engineering, National University of Singapore, Singapore, 117574 Singapore

**Keywords:** Thermocell, Thermosensitive crystallization, Porous carbon electrode, Power density, Low-grade heat harvest

## Abstract

Thermocells are garnering increasing attention as a promising thermoelectric technology for harvesting low-grade heat. However, their performance is often limited by the scarcity of high-performance redox couples that possess both high thermopower and rapid redox kinetics. This work addresses this challenge by leveraging our recently developed copper (I/II) (Cu^+^/Cu^2+^) redox couple. We significantly enhance the performance of Cu-based liquid thermocells by integrating a thermosensitive crystallization process with etched carbon cloth electrodes, achieving synergistic improvements in thermodynamic and kinetic performance. The thermosensitive crystallization process establishes a persistent Cu^2+^ concentration gradient, boosting the thermopower from 1.47 to 2.93 mV K^−1^. Moreover, the etched carbon cloth electrodes provide a larger electroactive surface area and demonstrate a higher current density. Consequently, the optimized Cu^+^/Cu^2+^ system achieved an exceptional normalized power density *P*_max_ (*ΔT*)^−2^ of 3.97 mW m^‒2^ K^−2^. A thermocell module comprised of 20 cells directly power various electronic devices at a temperature difference of 40 K. This work successfully exhibits potential of Cu^+^/Cu^2+^ redox couple in thermoelectric conversion and introduces a valuable redox couple for high-performance thermocells.

**Supplementary Information:**

The online version contains supplementary material available at 10.1007/s40820-025-01977-w.

## Highlights

- A novel Cu^+^/Cu^2+^ redox couple was introduced to enable a thermosensitive crystallization process, significantly enhancing thermopower from 1.47 to 2.93 mV K^‒1^.
- A readily fabricated etched carbon cloth electrode offered an enlarged electroactive surface area, demonstrating superior current density through improved kinetics.
- The optimized Cu^+^/Cu^2+^ system, achieved through synergistic enhancements in thermodynamic and kinetic performance, delivered an outstanding normalized power density *P*_max_ (*ΔT*)^‒2^ of 3.97 mW m^‒2^ K^‒2^.

**Supplementary Information:**

The online version contains supplementary material available at 10.1007/s40820-025-01977-w.

## Graphical abstract

**Figure:**
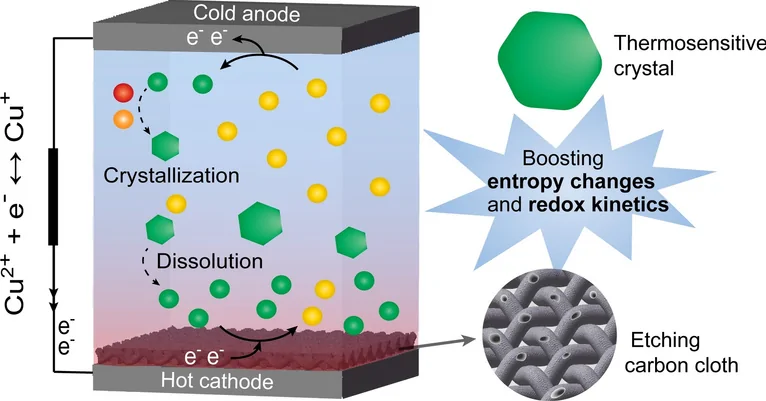

## Introduction

As global energy demands continue to rise and the search for sustainable energy solutions intensifies, the efficient utilization of available thermal energy resources, especially those that are often neglected, becomes increasingly critical [1, 2]. Low-grade heat, defined as thermal energy below 100 °C, is an often-overlooked and underutilized resource. It is abundantly available in various environmental sources, including geothermal and solar-thermal energy, as well as waste heat generated by power plants, data centers, and even the human body [3, 4]. Thermoelectric technologies, which convert this ubiquitous thermal energy directly into useful electricity, hold significant potential for powering low-energy smart devices within the Internet of Things (IoT) [5–7]. Among them, liquid thermocells (LTCs) present a promising new solution, offering high thermopower (in the mV K^−1^ range), low cost, and easy scalability. LTCs exploit the temperature dependence of electrochemical redox potentials, known as the thermogalvanic effect, to generate continuous electricity. The power output (*P* = *V* ∙ *J*) of LTCs depends on both thermodynamic and kinetic features. The thermodynamic aspect is characterized by thermopower *S*_e_, defined as *S*_e_ = $\partial V/\partial T = \Delta S/nF$, where *ΔS* represents the entropy change for the redox reaction, *n* is the number of electrons transferred, and *F* refers to Faraday’s constant. The kinetic aspect is directly reflected in the current density (*J*). Overall, the normalized power density (*P*_max_ (*ΔT*)^−2^) serves as a comprehensive metric for evaluating and comparing the performance of TLCs.

Since the introduction of the concept of LTCs in the nineteenth century, only a limited number of redox couples have demonstrated potential for thermoelectric conversion, including Fe(CN)_6_^3−^/Fe(CN)_6_^4−^ [8–10], Fe^2+^/Fe^3+^ [11, 12], Cu/Cu^2+^ [13, 14], I^−^/I_3_^−^ [15, 16], and others [17, 18]. Among these redox couple systems, the Fe(CN)_6_^3−^/Fe(CN)_6_^4−^ system stands out as the most preferred thermogalvanic system primarily due to its intrinsic characteristics. The pristine 0.4 M Fe(CN)_6_^3‒^/Fe(CN)_6_^4‒^ system exhibits a high *S*_e_ of approximately −1.4 mV K^–1^ and rapid redox kinetics, serving as a benchmark for LTC [19, 20]. Over the past decade, the field of LTCs has witnessed remarkable progress and impressive performance enhancements through advancements in electrolytes [10, 21–24], electrodes [25–27], and device architectures [28–30], particularly within the Fe(CN)_6_^3‒^/Fe(CN)_6_^4‒^ system. For instance, Duan et al. introduce strong chaotropic guanidinium cations and highly soluble urea into Fe(CN)_6_^3‒^/Fe(CN)_6_^4‒^ system, synergistically enlarging the entropy difference. This resulted in a high *S*_e_ of −4.2 mV K^–1^ and a normalized power density (*P*_max_ (*ΔT*)^−2^) of 1.1 mW K^−2^ m^−2^ [9]. Furthermore, carbon nanomaterials have emerged as cost-effective electrodes, providing high current densities in LTCs due to their increased surface area and rapid electron transfer kinetics [8, 31, 32]. For example, activated carbon cloth employed as electrodes in the Fe(CN)_6_^3‒^/Fe(CN)_6_^4‒^ system can achieve a *P*_max_ (*ΔT*)^−2^ of up to 1.80 mW K^−2^ m^−2^ [26]. In a holistic approach, Yu et al. pioneered a thermosensitive crystallization strategy in conjunction with porous carbon fiber electrodes, yielding a high *S*_e_ of −3.73 mV K^–1^ and a remarkable *P*_max_ (*ΔT*)^−2^ of 7.08 mW K^−2^ m^−2^ [28].

Conversely, other redox couple systems have struggled to exceed 2 mW K^−2^ m^−2^ due to intrinsic performance limitations. For example, applying similar thermosensitive crystallization and 3D multi-structured electrodes to enhance the thermodynamic and kinetic properties of the Cu/Cu^2+^ system yields a *S*_e_ of only 1.66 mV K^‒1^ and a *P*_max_ (*ΔT*)^−2^ of 0.71 mW K^−2^ m^−2^ [13]. Although the I^‒^/I_3_^‒^ system achieves an exceptionally high *S*ₑ of 9.62 mV K⁻^1^ through strong hydrophobic interactions between thermoresponsive methylcellulose and I_3_^‒^ ions, it only reaches a *P*_max_ (*ΔT*)^−2^ of 0.36 mW K^−2^ m^−2^ [15]. This is attributed to the slow kinetic rate inherent to this system. Although optimized systems show significant improvements over their unoptimized counterparts, they still fall short when compared to the Fe(CN)_6_^3‒^/Fe(CN)_6_^4‒^ system. The Fe^2+^/Fe^3+^ system displays a *S*_e_ that is highly dependent on the choice of counter ions, with the perchlorate system (Fe(ClO_4_)_2_/Fe(ClO_4_)_3_) yielding a relatively high *S*_e_ of ~1.65 mV K^–1^ [33, 34]. However, this system often requires expensive platinum electrodes to ensure fast kinetics and corrosion resistance. Utilizing highly catalytic electrodes in a cylindrical architecture, the Fe(ClO_4_)_2_/Fe(ClO_4_)_3_ system achieves a *P*_max_ (*ΔT*)^−2^ of 1.92 mW K^−2^ m^−2^ [35]. It is important to note that the advancement of high-performance LTCs is currently constrained by the challenge of discovering redox couples that exhibit both high *S*ₑ and rapid redox kinetics simultaneously.

**Fig. 1:**
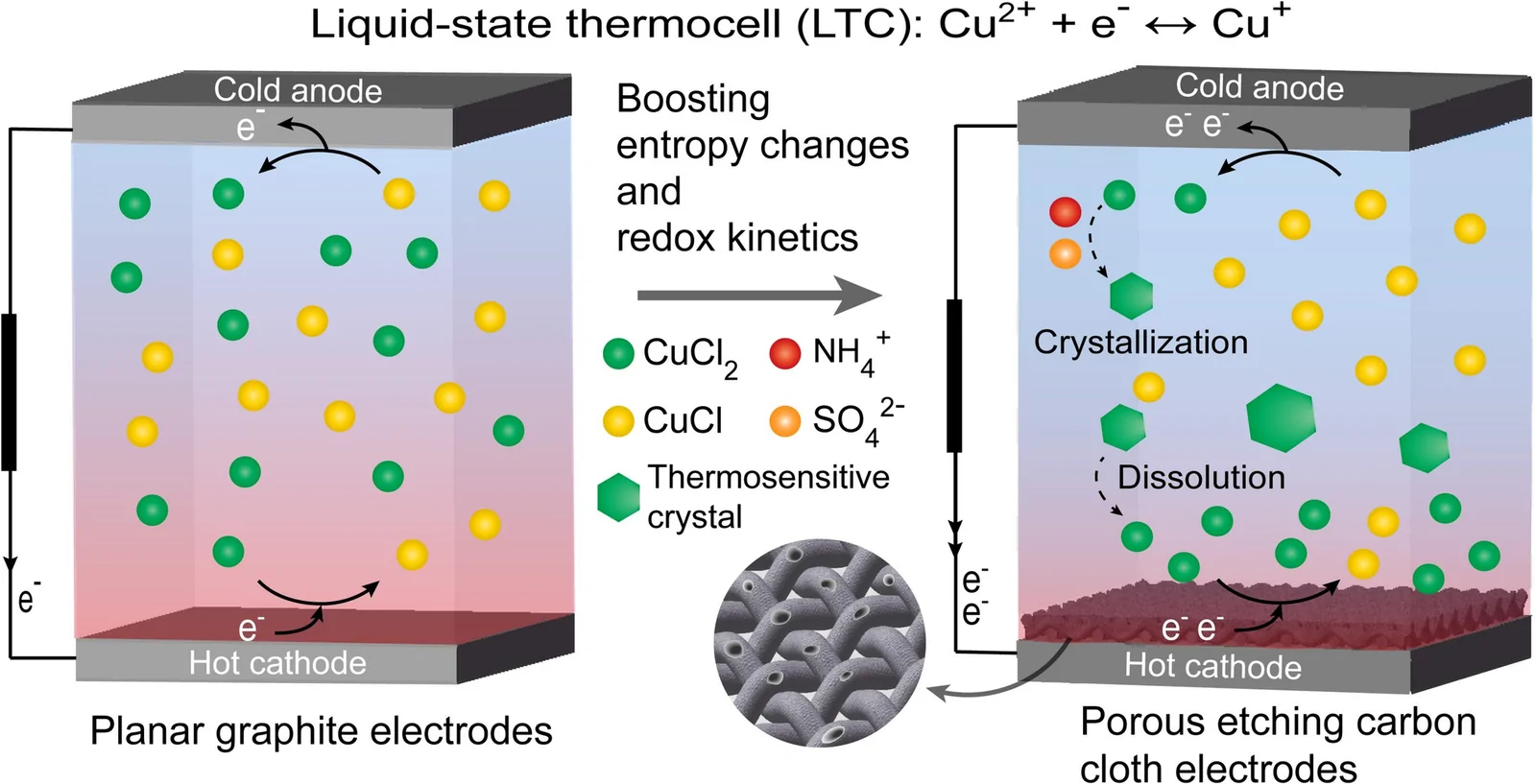
Schematic representation of a high-performance Cu-based liquid thermocell. This design incorporates a thermosensitive crystallization process and etched carbon cloth electrodes to synergistically enhance entropy changes and redox reaction kinetics. The reduction reaction (Cu^2+^ + e^–^ → Cu^+^) occurs at the hot electrode (cathode), and the oxidation reaction (Cu^+^ → Cu^2+^ + e.^–^) takes place at cold electrode (anode)

Recently, we have developed an innovative redox couple, Cu^+^/Cu^2+^ (Fig. 1, left), which demonstrate exceptional thermogalvanic performance. This system rivals the performance of the benchmark 0.4 M K_3_Fe(CN)_6_/K_4_Fe(CN)_6_ system, showcasing an intrinsically high *S*ₑ of 1.51 mV K^‒1^ and fast redox kinetics. In this work, we achieve further performance enhancements by combining a thermosensitive crystallization process with etched carbon cloth electrodes, enabling synergistic optimization of both thermodynamic and kinetic properties. The thermosensitive crystallization process, driven by ammonium sulfate, creates a sustained Cu^2+^ concentration gradient that contributes to a substantial increase in entropy change (*ΔS*), thereby boosting the *S*ₑ from 1.47 to 2.93 mV K^‒1^. Additionally, the etched carbon cloth electrodes are prepared via an easily accessible alkalization and annealing treatment, which increases hydrophilicity and surface area. This results in a larger electroactive surface area and consequently leads to greater current density. As a result, the optimized Cu^+^/Cu^2+^ system achieved an exceptionally high *P*_max_ (*ΔT*)^−2^ of 3.97 mW m^‒2^ K^‒2^. Given to the high *S*_e_ and *J* of Cu^+^/Cu^2+^ system, a prototype module consisting of 20 units generates an open-circuit voltage of 2.14 V and a maximum power output of 27.19 mW under a temperature difference (*ΔT*) of 40 K, which is sufficient to directly power low-energy power devices. This work successfully demonstrates the potential of the Cu^+^/Cu^2+^ redox couple in thermoelectric conversion and introduces a valuable new redox couple for high-performance thermocells.

## Experimental Section

### Materials

Cuprous chloride (CuCl), cupric chloride (CuCl_2_), potassium ferricyanide (K_3_Fe(CN)_6_), potassium ferrocyanide (K_4_Fe(CN)_6_), sodium sulfate (Na_2_SO_4_), potassium sulfate (K_2_SO_4_), ammonium sulfate ((NH_4_)_2_SO_4_), tetramethylammonium sulfate, and guanidinium sulfate were obtained from Shanghai Aladdin Co. Ltd. Hydrochloric acid (HCl) and ammonium chloride (NH_4_Cl) were purchased from the Chengdu Kelong Co. Ltd. All chemical reagents were used without further purification. Graphite sheets and carbon cloth were purchased from Longyao Carbon New Material Technology Co. Ltd. (China), respectively. The deionized water used in all experiments was prepared by an ATSro 10.

### Preparation of Electrodes, Electrolytes and LTCs Devices

3D porous carbon cloth electrodes were prepared by two steps. First, carbon cloth was immersed in 1 M KOH solution at 70 °C for 24 h, stirred at 500 rpm. Thereafter, carbon cloth was washed with deionized water several times and dried in vacuum at 60 °C for 12 h. Second, carbon cloth was annealed at 600 °C in a tube furnace. The annealing time affected surface structure of carbon cloth fiber. We set five time points: 0, 1, 3, 5, and 7 h. The etched carbon cloth was washed and placed in the oven to dry for use.

For Cu-based liquid thermocell, CuCl and CuCl_2_ were dissolved in this aqueous solution containing 0.1 M HCl and 0.9 M NH_4_Cl. The 0.15 M CuCl and 0.4 M CuCl_2_ dissolved in this aqueous solution were used as the pristine electrolyte for LTC. The optimized electrolyte was prepared by adding (NH_4_)_2_SO_4_ into the pristine electrolyte. Similarly, the electrolyte with other molarity ratios and additives was prepared for comparison. Typically, a planar single cell was used to demonstrate the thermoelectric performance of the LTC/TC-LTC. As shown in Fig. S1, the planar cell was assembled by four steps: first, attaching the carbon cloth electrodes and/or graphite sheets to the poly (methyl methacrylate) (PMMA) frame and sealing with epoxy glue; second, injecting electrolyte to fill up the cell; third, sealing the cell by the nano transparent double-sided tape; forth, horizontally placing the cell to test. The cross-sectional area of the frame was 3.24 cm^2^, and the height was 1.2 cm. The hot side was heated by an electrical heating plate, and the cold side was cooled by a thermoelectric cooler contacting a water-cooling plate. In all measurements, the cold electrode temperature is controlled at ~20 °C. A similar process was used to fabricate an integrated TC-LTC module (Fig. 5a). In brief, a plastic frame containing 20 isolated cells was filled with optimized electrolyte. Each isolated cell was the same size as the planar cell above. Finally, the isolated cells were connected in series by Cu wires.

### Material and Electrical Characterizations

The concentration of Cu^2+^ and Cu^+^ in the electrolytes was measured by UV–Vis spectro-photometry (Shimadzu UV-3600) with the loading buffer diluted ~350 times by 3 M NH_4_Cl solution. The crystal structure of samples was analyzed by X-ray diffraction (XRD, PANalytical X’Pert Powder). Fourier-transform infrared spectroscopy (FTIR) was performed using Thermo Fisher Scientific Nicolet iS50. X-ray photoelectron spectroscopy (XPS) was performed with an ESCALAB-250Xi X-ray photoelectron spectrometer. The solubility (defined as g per 100 g solution) of the crystals at different temperatures was directly measured. The dried samples were gradually added them to aqueous solution (contain 0.9 M NH_4_Cl and 0.1 M HCl) (5 g) at different temperatures and thermostatically incubated until the solution was saturated. The amounts of dried samples added were multiplied by 20 to obtain their solubility at different temperatures. The morphology of the various electrodes was characterized by a scanning electron microscope (SEM, JXA-8530F Plus). The contact angles of water droplets on the various electrodes were measured by a contact angle meter (SL200B, Kino) at room temperature.

Cyclic voltammetry (CV) scanning was performed at 10 mV s^−1^. The Randles–Sevcik equation is given by $I_{p} = 2.69 \times 10^{5} \cdot {\mathrm{ESA}} \cdot D^{1/2} \cdot n^{3/2} \cdot \nu^{1/2} \cdot C$, where *I*_*p*_ is the faradaic peak current, *n* is the number of electrons transferred during the redox reaction, ESA is the electroactive surface area, *C* is the concentration of the probe molecule, *ν* is the potential scan rate, and *D* is the diffusion coefficient. Electrochemical impedance spectra (EIS) measurements were taken in the frequency range between 10 kHz and 100 mHz. CV and EIS tests were performed on electrochemical workstation (Bio-Logic, VMP3, SN 0897). Voltage–time, current–voltage, and power–voltage curves were measured with a Keithley 2400 instrument, and the corresponding temperature profiles were recorded by a thermocouple data logger (USB-TC-08, Pico Technology, St. Neots). The current–voltage curves were recorded by the points measured from the open-circuit voltage to 0 V, and the power–voltage curves were calculated by the product of the corresponding current and voltage values.

## Results and Discussion

The LTC consists of an electrolyte containing a redox couple sandwiched between two electrodes (Fig. 1, left). The device architecture is straightforward and does not require complex manufacturing processes, demonstrating good reliability (Figs. S1 and S2). Our laboratory has developed a novel redox couple, Cu^+^/Cu^2+^, achieved by stabilizing cuprous ions in chloride-rich solutions. The solubility of CuCl/CuCl_2_ in an electrolyte solution of 0.9 M NH_4_Cl and 0.1 M HCl can reach up to 0.15 M, yielding an inherent *S*_e_ as high as 1.51 mV K^–1^ (Fig. S3). This promising pristine system presents significant opportunities for further improvement. As detailed in Note S1, the potential difference under a temperature difference (also referred to as *S*_e_) depends on both the solvent-dependent entropy difference (*ΔS*) between the redox couple and the concentration ratio difference (*ΔC*_*r*_) between the hot and cold sides of the LTC. Typically, the concentration gradient is thermodynamically unstable and tend to spontaneously decays into a homogeneous stable state (Fig. 1, left), where *ΔC*_*r*_ equals zero [13, 28]. Consequently, the *S*_e_ for the traditional LTC is driven solely by *ΔS*. The *ΔS* can be increased through the addition of specific organic solvents or specific additives [9, 36]. However, their role is single and they may have minimal or even opposing impacts on the improvement of redox kinetics within the LTC [36, 37]. Yu et al*.* proposed a thermosensitive crystallization strategy that enhances *S*_e_ through the contributions of both *ΔS* and *ΔC*_*r*_ [28]. In the thermodynamically stable state, a concentration gradient is established within LTC, which not only enhances *S*_e_ but also effectively suppresses thermal conductivity without scarifying kinetic performance.

To further boost *S*_e_, we establish a Cu^2+^ concentration gradient in CuCl/CuCl_2_ solution using the ammonium sulfate ((NH_4_)_2_SO_4_) to induce thermosensitive crystallization (Fig. 1, right). Specifically, the addition of (NH_4_)_2_SO_4_ preferentially binds Cu^2+^ to form thermosensitive crystals on the cold side (top), where the crystals precipitate due to gravity and subsequently redissolve on the hot side (bottom). This process creates a Cu^2+^ concentration gradient, with lower concentration near the cold electrode and higher concentration near the hot electrode. We refer to this system as a thermosensitive crystallization-boosted LTC (TC-LTC). However, the relatively low concentration of the redox couple (0.15 M CuCl/CuCl_2_) yields fewer crystals (Fig. S4). For effective thermosensitive crystallization and dissolution, sufficient crystal formation is essential. Therefore, we re-optimize the pristine LTC system, i.e., the system without thermosensitive crystallization. As the solubility of CuCl has reached its maximum under the given solvent conditions, we opted to increase only the concentration of CuCl_2_ during the re-optimization process. By increasing the CuCl_2_ concentration, we can enhance the number of crystals formed. When the concentration of CuCl_2_ is increased from 0.15 to 0.4 M, the *S*_e_ of the system reaches its maximum (Fig. S4). The optimal composition for the pristine LTC is determined to be 0.15 M CuCl/0.4 M CuCl_2_. The pristine LTC not only has a high *S*_e_ of 1.47 mV K^−1^ but also exhibits fast rapid kinetics, which are comparable to those of the 0.4 M Fe(CN)_6_^3‒^/Fe(CN)_6_^4‒^ benchmark system (Figs. S2, S5, S6). Moreover, the short-circuit current density (*J*_sc_) does not fluctuate significantly over 12 h at a temperature difference (*ΔT*) of 40 K, indicating excellent stability for the pristine system.

**Fig. 2:**
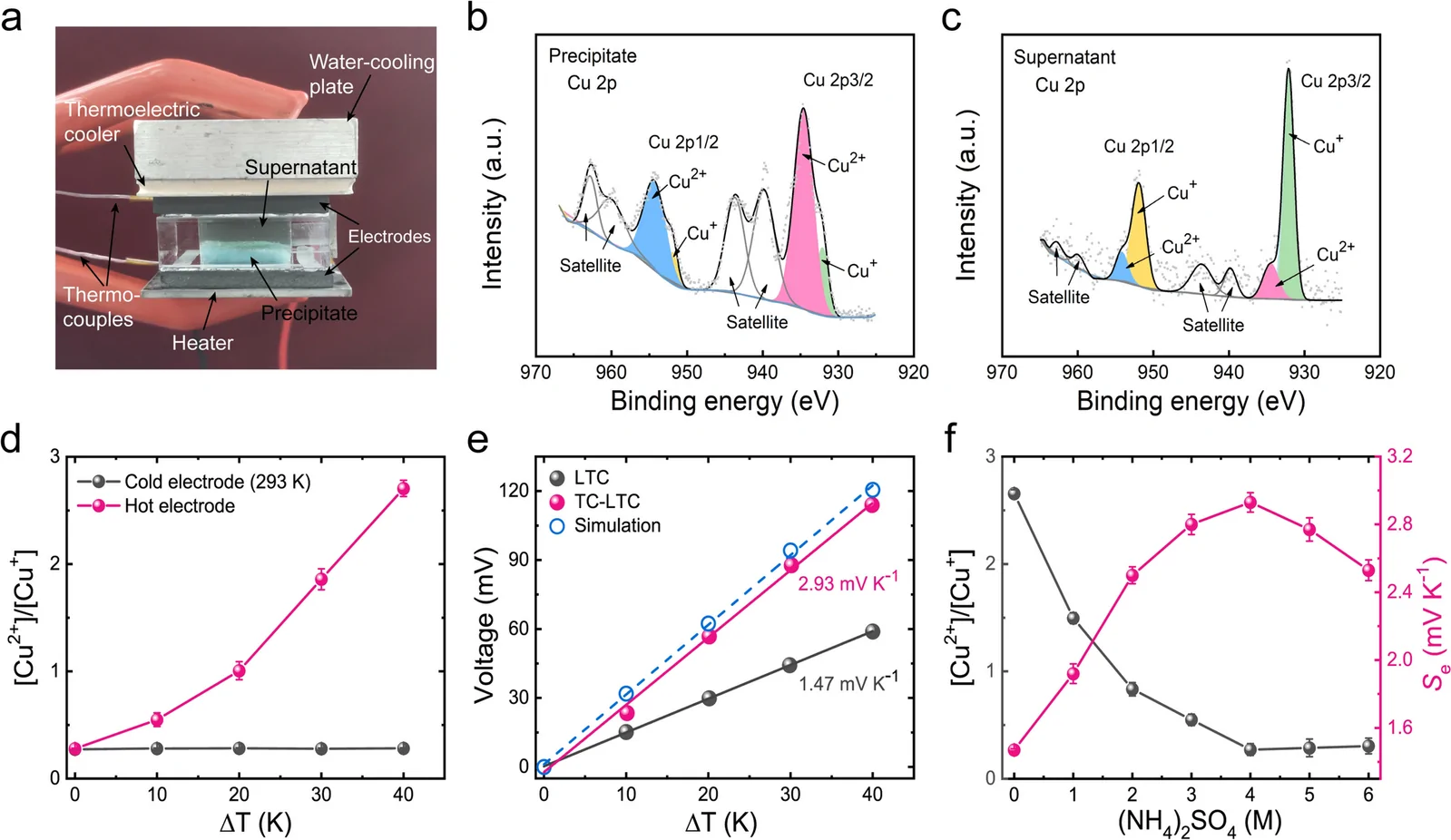
Crystallization-inducing enhancement of the *S*_e_ in the TC-LTC. **a** Photograph of a single planar TC-LTC cell. The water-cooling plate and thermoelectric cooler work together to maintain the cold side temperature at 293 K. Cu 2*p* XPS spectra for dried powders from **b** the precipitate and **c** the supernatant in the (NH_4_)_2_SO_4_-added electrolytes. **d** [Cu^2+^]/[Cu^+^] concentration ratio at the cold and hot electrodes as a function of the temperature difference (*ΔT*). **e** Open-circuit voltage (*V*_oc_) of the LTC and TC-LTC at different *ΔT* values, with simulated results (dashed line) aligning with experimental data. **f** [Cu^2+^]/[Cu^+^] concentration ratio in the electrolyte at 293 K and corresponding *S*_e_ values with the addition of (NH_4_)_2_SO_4_ at different concentrations

The addition of (NH_4_)_2_SO_4_ causes a color change in the CuCl/CuCl_2_ solution, shifting from dark green to light blue, as blue crystals form (Figs. 2a and S7). The light blue supernatant suggests a decrease in Cu^2+^ concentration. To further confirm the specific binding of (NH_4_)_2_SO_4_ to Cu^2+^, X-ray photoelectron spectroscopy (XPS) analysis is used (Fig. 2b, c). The peaks at 954.2 eV and 934.4 eV correspond to Cu(II) 2*p*_1/2_ and Cu(II) 2*p*_3/2_, while the peaks at 952.0 eV and 932.1 eV are assigned to Cu(I) 2*p*_1/2_ and Cu(I) 2*p*_3/2_ [38]. These results confirm that Cu^2+^ predominantly resides within the crystals, whereas Cu^+^ is primarily found in the supernatant. Additionally, this interaction between ammonium (${\mathrm{NH}}_{4}^{+}$) and Cu^2+^ in the crystals is supported by Fourier-transform infrared spectroscopy (FTIR) (Fig. S8).

The thermosensitive crystals, forming at the cold end and dissolving at the hot end, induce a copper ion concentration gradient, creating a *ΔC*_*r*_. We measure the concentration ratio profile of [Cu^2+^]/[Cu^+^] in the TC-LTC under varying *ΔT* from UV–Vis absorption spectra (Figs. 2d, S9–S11, and Note S2). At the cold side (293 K), nearly complete crystallization of Cu^2+^ led to low [Cu^2+^]/[Cu^+^] ratio of ~0.27. In contrast, as the temperature increased on the hot side, rapid dissolution of the crystals resulted in a [Cu^2+^]/[Cu^+^] ratio of 2.66 at 333 K. This creates a substantial *ΔC*_*r*_ between the electrodes, which rises with increasing *ΔT*. Given that *S*_e_ is synergistically influenced by both *ΔS* and *ΔC*_*r*_, the voltage output of the TC-LTC shows significant enhancement (Figs. 2e and S12). The experimentally measured voltage values agree with those simulated using Eq. S6 and the measured [Cu^2+^]/[Cu^+^] ratios. The maximum *S*_e_ achieved is 2.93 mV K^–1^, which is nearly double that of the original LTC system (1.47 mV K^–1^). Further optimization of the (NH_4_)_2_SO_4_ addition (Figs. 2f, and S13, S14) indicates that a concentration of 4 M achieves complete crystallization of Cu^2+^ at lower temperatures, leading to the largest concentration gradient and thus maximizing *S*_e_. Higher (NH_4_)_2_SO_4_ concentrations result in decreased *ΔC*_*r*_ due to concomitant crystallization of Cu⁺, which ultimately leads to a reduction in *S*_e_.

**Fig. 3:**
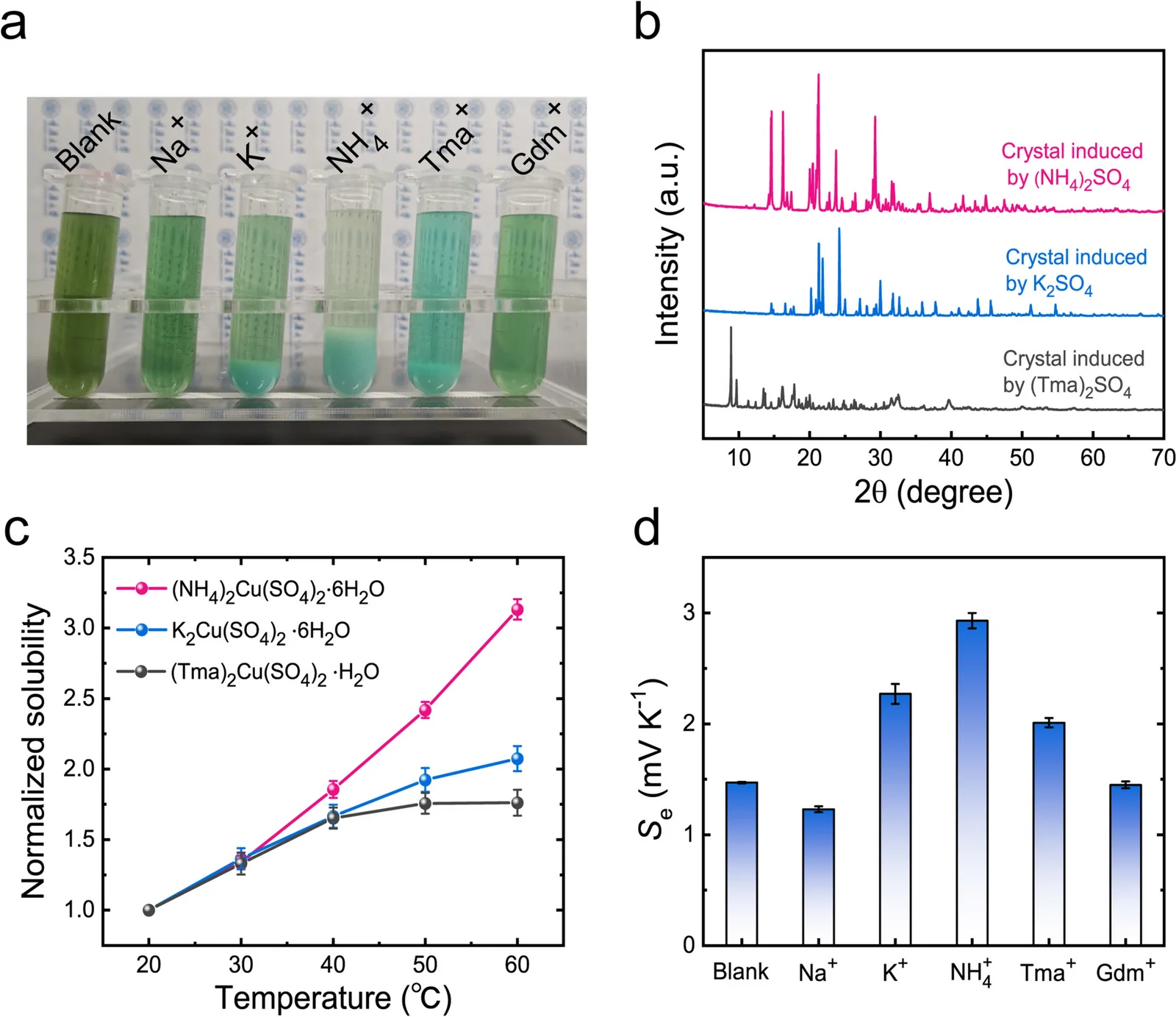
Various additives for inducing thermosensitive crystallization and enhancing *S*_e_.** a** Photographs of the electrolyte with various additives at 293 K. **b** XRD patterns of the crystals induced by various additives, confirming their identities as (NH_4_)_2_Cu(SO_4_)_2_·6H_2_O, K_2_Cu(SO_4_)_2_·6H_2_O and (Tma)_2_Cu(SO_4_)_2_·H_2_O, respectively. **c** Normalized solubility of the three crystals as a function of temperature. **d** Comparison of *S*_e_ values for the thermocell using various additives

To illustrate the underlying mechanisms of thermosensitive crystallization, we systematically investigate various monovalent cation sulfates as additives. The ability to induce crystallization is observed only with potassium (K^+^), ammonium (${\mathrm{NH}}_{4}^{+}$) and tetramethylammonium (Tma^+^) (Figs. 3a and S15). Subsequently, we examine the crystal structure using XRD (Fig. 3b). The crystals induced by (NH_4_)_2_SO_4_, K_2_SO_4_, and (Tma)_2_SO_4_ are identified as (NH_4_)_2_Cu(SO_4_)_2_∙6H_2_O, K_2_Cu(SO_4_)_2_∙6H_2_O, and (Tma)_2_Cu(SO_4_)_2_∙H_2_O, respectively [13, 39]. These double sulfates of divalent metals with various monovalent cations could be effectively explained based on the hard–soft acid–base (HSAB) principle, which states that hard Lewis acids preferentially bind with hard Lewis bases and soft Lewis acids with soft Lewis bases [40, 41]. In order to co-crystallize, the complex cation and the complex anion must have similar acid and base strengths [42]. As a typical intermediate hardness cation, Cu^2+^ is considered to coordinate with either H_2_O molecules or anions during the formation of the double salts, depending on the relative proportions of H_2_O and anions and the hardness of the second cation [13]. In the pristine electrolyte solution (0.9 M NH_4_Cl and 0.1 M HCl), Cl⁻ progressively replaces water molecules in the hydrated [Cu(H_2_O)_6_]^2+^ complex, forming cupric-chloro complexes such as [CuCl(H_2_O)_5_]^+^ and [CuCl_2_(H_2_O)_4_]^0^ [43]. However, upon the introduction of sulfates, the increased sulfate concentration (4 M) promotes coordination between Cu^2+^ and ${\mathrm{SO}}_{4}^{{2{-}}}$, facilitating the formation of a softer complex anion. Moreover, the harder second cations Li^+^, Na^+^ and guanidinium (Gdm^+^) do not induce Cu^2+^ crystallization, likely due to unfavorable HSAB interactions. In contrast, the softer second cations ${\mathrm{NH}}_{4}^{+}$, K^+^, and Tma^+^ could potentially form double salts with softer complex anion composed of with Cu^2+^ and ${\mathrm{SO}}_{4}^{{2{-}}}$.

The thermosensitive solubility of Cu^2+^-associated crystals is a critical for achieving a high *S*_e_. Investigation of temperature-dependent solubility reveals that the (NH_4_)_2_SO_4_-induced crystals exhibit the highest thermosensitive solubility, resulting in the greatest concentration difference with increasing temperature (Figs. 3c and S16, S17). Thermodynamic theory, captured by the equation *ΔG* = *ΔH*–*TΔS*, explains the differences in thermosensitivity among the crystals. Generally, crystals that undergo significant entropy changes (*ΔS*) combined with small enthalpy changes (*ΔH*) upon dissolution lead to a notable decrease in Gibbs free energy (*ΔG*) with a slightly increase in temperature, resulting in high thermosensitivity [28]. The crystal induced by (NH_4_)_2_SO_4_ contains six hydrated water molecules and features the relatively large ${\mathrm{NH}}_{4}^{+}$ cation, resulting in the highest structural complexity and a large *ΔS*. This highly hydrated crystal is less tightly bound and exhibits low lattice energy [44], which translates to a small Δ*H* during dissolution. Consequently, this unique characteristic contributes to the highest *S*_e_ observed in the (NH_4_)_2_SO_4_-enhanced TC-LTC system (Figs. 3d and S18).

The power output of the LTCs is influenced not only by the thermodynamic parameter *S*_e_, but also by the current density [8]. Electrodes play a significant role in current delivery, particularly the microstructures of the electrodes [26, 45]. Initial experiments utilize a cost-effective graphite plate; however, its planar structure and limited specific surface area restrict the kinetic rates of the thermocell. In contrast, porous carbon electrodes offer a larger specific surface area, providing more active sites for redox reactions per unit volume, which contributes to a higher current density [46, 47]. Carbon cloth, known for its large specific surface area, is widely employed as an electrode material in the fields of energy storage and conversion [48–50]. To further enhance the specific surface area, an etched carbon cloth electrode is prepared through alkalization treatment followed by annealing at 600 °C (Fig. S19). The alkalization treatment involves soaking the carbon cloth in a 1 M KOH solution at 70 °C, which improves its hydrophilicity (Fig. S20). This enhancement facilitates easier access of the aqueous electrolyte to the carbon cloth, likely due to the formation of oxygen-containing functional groups [51, 52]. The alkalized carbon cloth electrode is designated as CC-Alkali. During the subsequent annealing process, residual KOH in the carbon cloth etches the surfaces of the carbon fibers, creating numerous tiny pores and further increasing the specific surface area [53]. The resulting electrode is referred to as CC-Alkali+A. Details of the etching mechanism are provided in Note S3. As the annealing time is extended, the density of surface micropores gradually increases (Figs. S21 and S22). However, when the annealing time reaches 7 h, the fibers exhibit ulceration, which leads to fiber breakage (Fig. S23).

**Fig. 4:**
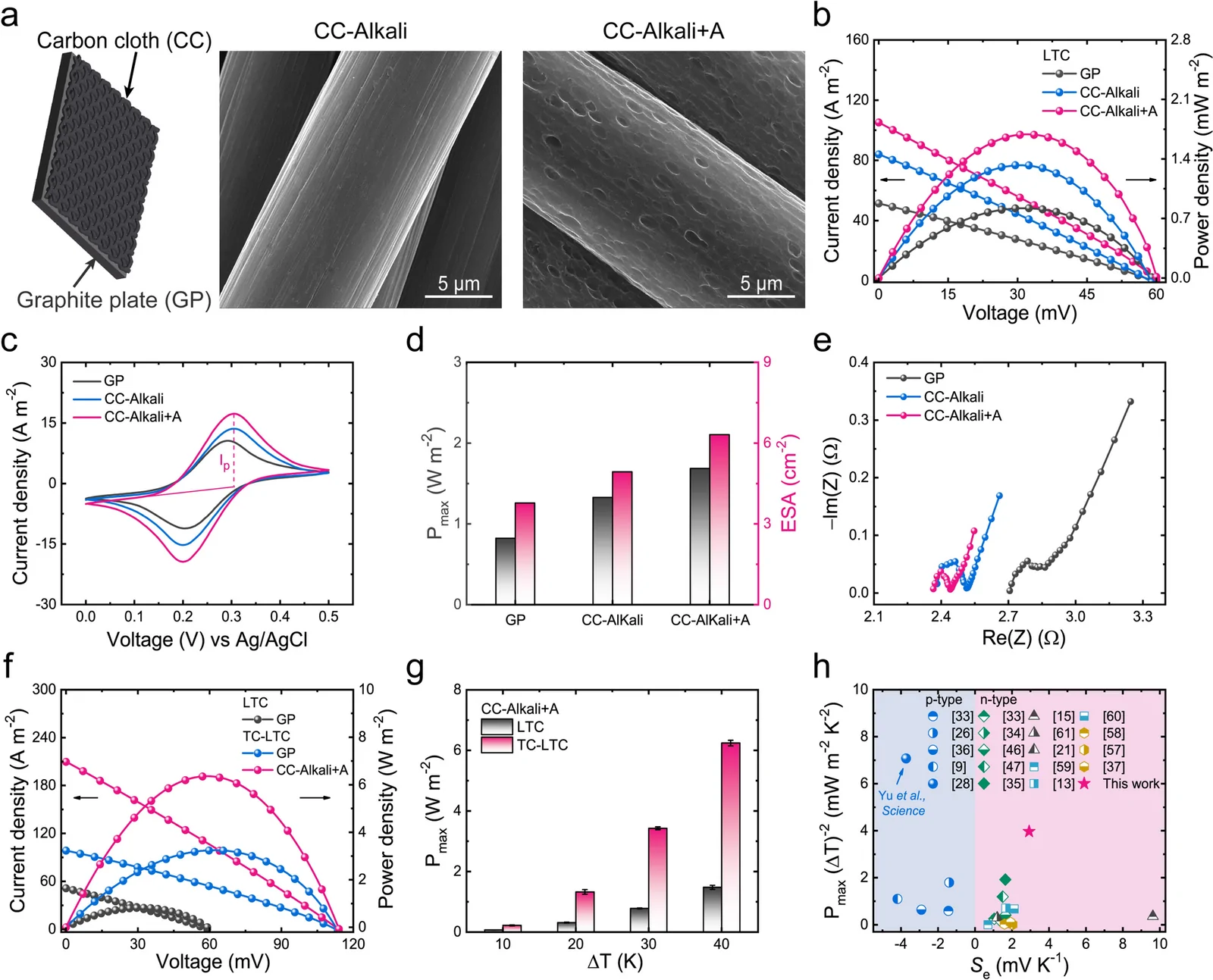
Electrode optimization of the Cu-based thermocells. **a** Diagram of electrode structure along with SEM images of the surface of carbon cloth. The alkalized carbon cloth electrode is labeled as CC-Alkali, while the etched carbon cloth electrode, produced through alkalization and annealing treatment, is labeled as CC-Alkali+A. **b** Current–voltage and power–voltage curves for the LTC using different electrodes at a *ΔT* of 40 K. **c** Cyclic voltammograms of different electrodes. **d** Maximum output power density *P*_max_ of LTC with different electrodes and electroactive surface area (ESA) values. **e** Electrochemical impedance spectra of different electrodes. **f** Current–voltage and power–voltage curves for the LTC and TC-LTC using different electrodes at a *ΔT* of 40 K. **g** Maximum power density (*P*_max_) for the LTC and TC-LTC utilizing CC-Alkali+A electrodes at varying *ΔT*. **h** Comparison of *P*_max_ (*ΔT*)^‒2^ and *S*_e_ values for this work and the other LTCs

Subsequently, we assembled LTC and TC-LTC devices using the electrodes described above (Fig. 4a). In brief, a plastic cell is filled with an electrolyte and sealed with treated carbon cloth, followed by graphite plates (GPs), which serve as the electrodes. Based on the assessments of current output of the LTC, we optimized the annealing time to 5 h (Fig. S24). A comparison of different types of electrodes used in the LTC is presented in Fig. 4b. The *J*_sc_ of the LTC utilizing the CC-Alkali+A electrodes reaches 105.23 A m^−2^, which is greater than that of the LTC with GP electrodes or CC-Alkali electrodes. Correspondingly, maximum power density *P*_max_ of this LTC is significantly increased. To understand this enhancement, we leverage the electroactive surface area (ESA), defined as the fraction of the electrode surface that is electrochemically active and participates in Faradaic reactions. The ESA provides a direct measure of the number of active sites and is thus a more relevant metric for electrochemical performance than geometric area [54]. Experimentally, the ESA could be determined from the peak current density (*I*_*p*_) in the cyclic voltammograms (CVs) of the electrodes (Fig. 4c). According to the Randles–Sevcik equation [55], a high faradaic peak current of an electrode exhibiting reversible kinetics indicates a high ESA [8, 56]. The ESAs of the GP, CC-Alkali, and CC-Alkali+A electrodes are estimated to be 3.77, 4.93, and 6.31 cm^2^, respectively. Generally, a higher ESA corresponds to a higher *P*_max_ (Fig. 4d). Furthermore, the CC-Alkali+A electrode also demonstrates a lower charge transfer resistance (*R*_ct_, represented by the diameter of the semicircle in a Nyquist plot) (Fig. 4e), which facilitates improved charge transport.

When utilizing the same GP electrodes, both the *S*_e_ and *J* of the TC-LTC system are enhanced compared to the LTC system (Fig. 4f). This increase in current density is attributed to the thermosensitive crystallization process. In TC-LTC system, a low local concentration of Cu^2+^ near the cold electrode promotes the oxidation reaction (Cu^+^ → Cu^2+^ + e^‒^), while a high local concentration of Cu^2+^ near the hot electrode enhances the reduction reaction of (Cu^2+^ + e^‒^ → Cu^+^). The cell employing CC-Alkali+A electrodes in the TC-LTC, which represents an optimized electrolyte and electrode system, achieves a *P*_max_ of 6.35 W m^‒2^ at a *ΔT* of 40 K. This is 7.74 times greater than that of the LTC system using GP electrodes (0.82 W m^‒2^) and 1.97 times greater than that of the TC-LTC system with GP electrodes (3.23 W m^‒2^), as shown in Fig. 4f. Additionally, the TC-LTC displays remarkable enhancements in output compared to the LTC with the same CC-Alkali+A electrodes across various ΔT values (Figs. 4g and S25). The *P*_max_ (*ΔT*)^−2^ of our optimized system reaches 3.97 mW m^‒2^ K^‒2^, remarkably surpassing those of current n-type LTCs, including [Co(bpy)_3_]^2+^/[Co(bpy)_3_]^3+^ system [37, 57, 58], Cu/Cu^2+^ system [13, 59, 60], I^‒^/I_3_^‒^ system [15, 21, 61], and various Fe^2+^/Fe^3+^ systems [33–35, 46, 47] (Fig. 4h and Table S1). In comparison to p-type Fe(CN)_6_^3‒^/Fe(CN)_6_^4‒^ systems, our optimized system outperforms most Fe(CN)_6_^3‒^/Fe(CN)_6_^4‒^-based LTCs [9, 26, 33, 36], except for the one reported by Yu et al*.* [28]. This motivates us to explore more suitable additives for thermosensitive crystallization to achieve higher *S*ₑ values and to develop more effective electrode optimization strategies for faster reaction kinetics. Moreover, we have evaluated the cost-performance metric (CPM) of various thermoelectric systems by considering the prices of raw materials. Compared to inorganic solid-state thermoelectric cells (ITECs) and organic solid-state thermoelectric cells (OTECs), our TC-LTC system demonstrates a potentially more cost-effective profile, as detailed in Table S2.

**Fig. 5:**
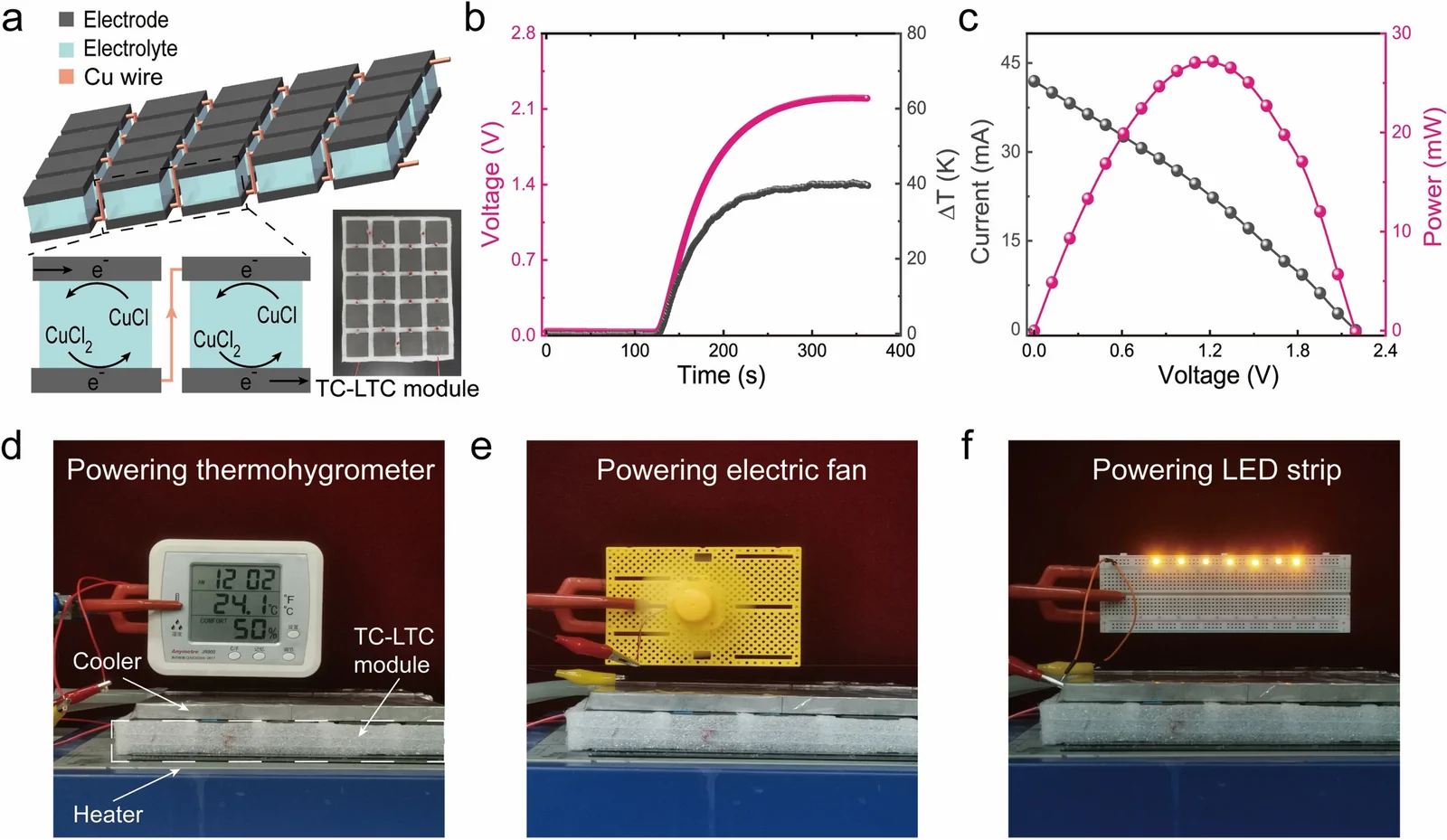
Electricity generation and demonstration of using a TC-LTC module to power electronic devices. **a** Schematic representation and photograph of a TC-LTC module containing 20 units connected in series. **b** Real-time voltage curves of the module with increasing temperature difference (*ΔT*). **c** Current–voltage curve and corresponding power output of the module at *ΔT* = 40 K. Image of the module directly powering various small electronic devices, including **d** a thermohygrometer, **e** an electric fan, and **f** an LED strip

Finally, we design a TC-LTC module by connecting 20 cells to demonstrate the viability for scale-up (Fig. 5a). Each isolated cell maintains the same electrode configuration and size as the planar cells tested earlier, and the isolated cells are connected in series by Cu wires. The module generates an open-circuit voltage (*V*_oc_) of 2.14 V and a short-circuit current of 41.95 mA, resulting in a *P*_max_ of 27.19 mW under a *ΔT* of 40 K (Fig. 5b, c). Relative to a single TC-LTC, the loss of current is attributed to increased internal resistance from the series connections [33]. Given the considerable power output, the module is capable of directly driving various electronic devices, including a thermohygrometer, an electric fan, and a light-emitting diode (LED) strip (Fig. 5d–f, and Movie S1). This suggests that the Cu-based TC-LTC we developed shows excellent potential for recovering low-grade heat.

## Conclusions

In summary, we have achieved a high-performance LTC through the synergistic thermodynamic and kinetic engineering of a novel Cu^+^/Cu^2+^ system, resulting in a high *S*_e_ of 2.93 mV K^‒1^ and a power density of 6.35 W m^‒2^ at *ΔT* of 40 K. Specifically, we developed a thermosensitive crystal, (NH_4_)_2_Cu(SO_4_)_2_·6H_2_O, induced by ammonium sulfate, which enables both a significant entropy change to enhance *S*_e_ and a concentration gradient simultaneously to improve the redox reaction of Cu^+^/Cu^2+^. Furthermore, we design an etched carbon cloth electrode with high hydrophilicity and a large electroactive area to further boost the redox kinetics. Consequently, our optimized Cu^+^/Cu^2+^-based LTC achieves a remarkable *P*_max_ (*ΔT*)^−2^ of 3.97 mW m^‒2^ K^‒2^, representing the best performance among current n-type LTCs. A prototype module consisting of 20 units successfully generates usable electrical energy, capable of directly powering small electronics, demonstrating the potential of this system for efficient low-grade heat harvesting. Prospectively, we will further enhance the kinetic properties of the Cu^+^/Cu^2+^ system by optimizing electrode materials and structures, as well as increasing the concentration of CuCl/CuCl_2_, to enable highly efficient p–n integrated devices with thermosensitive crystallization processes.

## Supplementary Information

Below is the link to the electronic supplementary material.

Supplementary file1 (DOCX 5754 kb)

Supplementary file2 (MP4 2556 kb)
