# Supplementary material for: High-Performance Cu-Based Liquid Thermocells Enabled by Thermosensitive Crystallization and Etched Carbon Cloth Electrode
Source: Nanomicro Lett. 2026 Jan 5;18:131. doi: 10.1007/s40820-025-01977-w (PMC12765770; doi:10.1007/s40820-025-01977-w)
Supplement: Supplementary file 1 — Supplementary file1 (DOCX 5754 kb) [file 40820_2025_1977_MOESM1_ESM.docx]

Supporting Information for

**High-Performance Cu-Based Liquid Thermocells Enabled by Thermosensitive Crystallization and Etched Carbon Cloth Electrode**

Wei Fang^1^, Zeping Ou^1^, Yifan Wang^1^, Zhe Li^1^, Qian Huang^1^, Pengchi Zhang ^1^, Xinzhe Li^1^, Yujie Zheng^1^, Lijun Hu^2^, Chen Li^1,3^, Jianyong Ouyang^4^, Kuan Sun^1^*

^1^ MOE Key Laboratory of Low-grade Energy Utilization Technologies and Systems, School of Energy & Power Engineering, Chongqing University, Chongqing 400044, P. R. China

^2^ Hunan Key Laboratory for the Design and Application of Actinide Complexes, School of Chemistry and Chemical Engineering, University of South China, Hengyang, Hunan 421001, P. R. China

^3^ Sibley School of Mechanical and Aerospace Engineering, Cornell University, Ithaca, New York 14853, United States

^4^ Department of Materials Science & Engineering, National University of Singapore, Singapore 117574, Singapore

*Corresponding author. E-mail: [kuan.sun@cqu.edu.cn](mailto:kuan.sun@cqu.edu.cn) (Kuan Sun)

Note S1 Theoretical analysis of the *S*_e_ for the Cu-based thermocell

For a liquid thermocell using CuCl/CuCl_2_ as redox couple, the reversible reactions can be symbolically described as follows:

$\text{Cu}^{\text{2+}}\text{ }\text{+}{\text{ }\text{e}}^{\text{‒}}\text{ }\text{↔}\text{ }\text{Cu}^{\text{+}}\text{ }\left( \text{S1} \right)$

When a temperature difference (ΔT) is applied to this LTC, the resulting electrochemical redox potential difference (ΔE) drives the aforementioned reaction to spontaneously occur in the reverse direction, generating a cell voltage (ΔV) and allowing electrons to flow through the external circuit. This phenomenon is commonly referred to as the thermogalvanic effect. The thermopower *S*_e_ represents the dependence of the potential difference (ΔE) on the temperature difference (ΔT). Thus, *S*_e_ can be expressed as [S1, S2]:

$$\text{S}_{\text{e}}\text{ }\text{=}\text{ }\frac{\text{∆V}}{\text{∆T}}\text{ }\text{=}\text{ }\frac{\text{∆}\text{S}}{\text{nF}}\text{ }\left( \text{S2} \right)$$

where Δ*S* is the entropy change for the redox reaction, n is the number of electrons transferred in the redox reaction, and F is Faraday’s constant.

To enhance the *S*_e_ of this thermocell system, the Δ*S* must be highly boosted. In general, the Δ*S* only takes into account the solvent-structural entropy of Cu^+^ and Cu^2+^. Besides, the concentration ratio difference (ΔC_r_) of [Cu^2+^]/[Cu^+^] also can lead to a Δ*S*, but its value is generally nearly zero in a thermodynamically stable state. Nonetheless, Zhou's work offered a novel approach by introducing a thermosensitive crystallization process, which effectively regulates the concentration of the targeted ions through temperature control [S3, S4]. Here, we analyze the potential for enhancing the *S*_e_ by regulating the ΔC_r_ of [Cu^2+^]/[Cu^+^].

According to the Nernst equation, the equilibrium potential (E) of the abovementioned redox reaction can be expressed as:

$$\text{E}\text{ }\text{=}{\text{ }\text{E}}^{\text{0}}\text{ }\text{+}\text{ }\frac{\text{RT}}{\text{nF}}\ln\frac{\text{α}_{\text{Cu}^{\text{2+}}}}{\text{α}_{\text{Cu}^{\text{+}}}}\text{ }\left( \text{S3} \right)$$

where E^0^ is the standard potential, and R is the ideal gas constant. The activity (αi) is defined as the product of the activity coefficient (γ_i_) and concentration ([i]) (α_i_ = γ_i_×[i]). Thus, Eq. (S3) can be written:

$$\text{E}\text{ }\text{=}{\text{ }\text{E}}^{\text{0}}\text{ }\text{+}\text{ }\frac{\text{RT}}{\text{nF}} \left[ \ln\frac{\text{γ}_{\text{Cu}^{\text{2+}}}}{\text{γ}_{\text{Cu}^{\text{+}}}}\text{ }\text{+}\ln\frac{\left[ \text{Cu}^{\text{2+}} \right]}{\left[ \text{Cu}^{\text{+}} \right]} \right]\text{ }\text{ }\text{ }\left( \text{S4} \right)$$

In general, *S*_e_ is calculated by:

$$\text{S}_{\text{e}}\text{ }\text{=}\text{ }\frac{\text{E}_{\text{hot}}\text{-}\text{E}_{\text{cold}}}{\text{T}_{\text{hot}}\text{-}\text{T}_{\text{cold}}}\text{ }\text{ }\text{ }\left( \text{S5} \right)$$

where the subscripts “hot” and “cold” indicate that the corresponding variables pertain to the hot and cold sides of the LTC, respectively. By substituting Eq. (S4) into Eq. (S5), S_e_ can be finally written as the sum of two terms:

$$\text{S}_{\text{e}}\text{=}\frac{\text{R}}{\text{F∆T}}\left[ \text{T}_{\text{hot}}\ln\frac{{\text{(}\text{γ}_{\text{Cu}^{\text{2+}}}\text{)}}_{\text{hot}}}{{\text{(}\text{γ}_{\text{Cu}^{\text{+}}}\text{)}}_{\text{hot}}}\text{-}\text{T}_{\text{cold}}\ln\frac{{\text{(}\text{γ}_{\text{Cu}^{\text{2+}}}\text{)}}_{\text{cold}}}{{\text{(}\text{γ}_{\text{Cu}^{\text{+}}}\text{)}}_{\text{cold}}} \right]\text{+}\frac{\text{R}}{\text{F∆T}}\left[ \text{T}_{\text{hot}}\ln\frac{\left[ \text{Cu}^{\text{2+}} \right]_{\text{hot}}}{\left[ \text{Cu}^{\text{+}} \right]_{\text{hot}}}\text{-}\text{T}_{\text{cold}}\ln\frac{\left[ \text{Cu}^{\text{2+}} \right]_{\text{cold}}}{\left[ \text{Cu}^{\text{+}} \right]_{\text{cold}}} \right]\text{ }\text{ }\left( \text{S6} \right)$$

whereas the first term containing the activity coefficient (γ) is considered to be dominated by the solvent-dependent difference in entropy between the redox couple; the second term is only related to the concentrations of Cu^+^ and Cu^2+^, which is defined as temperature-dependent concentration ratio difference ΔC_r_.

Generally, the concentration ratio [Cu^2+^]/[Cu^+^] is approximately consistent for the hot and cold sides, equaling to the initial concentration of the used electrolyte. Hence, the second term in Eq. (S6) is nearly zero. Clearly, if the concentration ratio [Cu^2+^]/[Cu^+^] at the hot side is much larger than that at the cold side, the value of the second term in Eq. (S6) could be highly boosted, and thereby contributed a giant *S*_e_. According to our experimental *S*_e_ value for the pristine electrolyte (Fig. 2e), the value of the first term in Eq. (S6) is approximately considered to be 1.47 mV K^–1^ in following calculations. We establish a Cu^2+^ concentration gradient using the ammonium sulfate (NH_4_)_2_SO_4_ to induce thermosensitive crystallization. Specifically, (NH_4_)_2_SO_4_ addition causes Cu^2+^ to crystallize on the cold side (top), precipitating due to gravity and subsequently redissolving on the hot side (bottom). This creates a Cu^2+^ concentration gradient, lower near the cold electrode and higher near the hot electrode (Fig. 1). As shown in the Figure S11, Supporting Information, we demonstrate the potential for regulating the open-circuit voltage (V_oc_, namely *S*_e_×ΔT) by the concentration ratio [Cu^2+^]/[Cu^+^] at the cold and hot side (*T*_h_ and *T*_c_ are 60 and 20 ℃, respectively). Furthermore, based on the measured concentration ratio [Cu^2+^]/[Cu^+^] at different temperature (Figure S10, Supporting Information), the simulated V_oc_ values for the TC-LTC are also calculated, exhibiting a near consistence with the experimental values (Fig. 2e).

Note S2 Analysis of cuprous and copper ion content by UV-vis spectra

In this work, the strategy of thermosensitive crystallization is adopted to enhance *S*_e_. Specifically, thermosensitive crystallization at the cold electrode and dissolution process at the hot electrode induce a persistent concentration gradient of redox ions by adding (NH_4_)_2_SO_4_. Therefore, the detection of Cu^2+^ ion and Cu^+^ ion content to analyze the change of concentration ratio [Cu^2+^]/[Cu^+^] is very critical. In 3 M Cl^‒^ environment, the absorption peak of Cu^+^ ion appears at 274 nm, while that of Cu^2+^ ion appears at 250 nm [S5] (Fig. S8). When the solution contains Cu^2+^ and Cu^+^ ions at the same time, there are two absorption peaks at 250 nm and 270 nm. The UV absorption spectra of 0.4 M CuCl_2_ and 0.15 M CuCl are superimposed in the range of 210~400 nm, which is similar to the UV absorption spectra containing both 0.4 M CuCl_2_ and 0.15 M CuCl in the solution (Fig. S8, dashed line). This suggests that the shift of Cu^+^ absorption peak in the solution containing Cu^2+^ is caused by the superposition of Cu^2+^ absorption band. Obviously, when the solution contains Cu^2+^ and Cu^+^ ions at the same time, the characteristic peak intensity of Cu^2+^ is inevitably affected by Cu^+^, and vice versa. Therefore, the content of Cu^2+^ and Cu^+^ ions cannot be calculated directly according to the intensity of characteristic peak.

According to Lambert-Beer's law, absorbance A = KCL, where K is the molar absorption coefficient; C is the concentration of detection solution; L is the thickness of the transmittance layer. Moreover, absorbance is additive [S6], at the same wavelength A = A_1_ + A_2_ + A_3_ + …… In order to resolve the concentration from the intensity at characteristic peak, we select the absorption intensity at 250 nm and 274 nm in the UV absorption spectra to analyze the copper ion content. Firstly, we obtain the UV absorption spectra of the solution with known Cu^2+^ (Cu^+^) concentration, and fit the relationship between the absorbance intensity at 250 nm and 274 nm and concentration of Cu^2+^ (Cu^+^) (Fig. S9). It can be seen that there is a good linearity between the absorbance intensity and concentration, with a correlation coefficient R of at least 0.998. Next, for unknown solutions containing X_1_ M CuCl and X_2_ M CuCl_2_, the absorbance of this UV absorption spectra at 250 nm and 274 nm is A_250_, A_274_, respectively. The Cu^2+^ and Cu^+^ ion concentration in solution can be obtained by solving the following binary first order equation:

$$\text{2.77 }\text{X}_{\text{1}}\text{ }\text{+}\text{ }\text{0.058}\text{ }\text{+}\text{ }\text{3.87 }\text{X}_{\text{2}}\text{ }\text{+}\text{ }\text{0.072}\text{ }\text{=}{\text{ }\text{A}}_{\text{250}}$$

$\text{7.93 }\text{X}_{\text{1}}\text{ }\text{+}\text{ }\text{0.018}\text{ }\text{+}\text{ }\text{2.52 }\text{X}_{\text{2}\text{ }}\text{+}\text{ }\text{0.054}\text{ }\text{=}{\text{ }\text{A}}_{\text{274}}$ (S7)

**Note S3 Mechanism of etching carbon cloth by KOH activation**

KOH activation method is widely used in broad fields to increase the specific surface area of carbon material. The activation mechanism has not been well understood because of the complexity due to the large number of variables in both the experimental parameters and the reactivity of different precursors used. In a general view, the reaction of carbon and KOH starts with solid–solid reactions and then proceeds via solid–liquid reactions including the reduction of potassium (K) compound to form metallic K [S7], the oxidation of carbon to form carbon oxide and carbonate, and other reactions among various active intermediates.

Based on previous research [S8-S10], three main activation mechanisms for KOH activation of carbon are concluded which have been widely accepted: (a) Etching the carbon framework by the redox reactions between various potassium compounds as chemical activating reagents with carbon as shown:

$$\text{6KOH}\text{ }\text{+}\text{ }\text{2C}\text{ }\text{→ 2K}\text{ }\text{+}\text{ }\text{3}\text{H}_{\text{2}}\text{ }\text{+}\text{ }\text{2}\text{K}_{\text{2}}\text{CO}_{\text{3}}\text{ }\text{ }\text{(S8)}$$

$$\text{K}_{\text{2}}\text{CO}_{\text{3}}\text{ }\text{+}\text{ }\text{2C}\text{ }\text{→}\text{ }\text{2K}\text{ }\text{+}\text{ }\text{3CO }\text{ }\left( \text{S9} \right)$$

$$\text{C}\text{ }\text{+}\text{ }\text{K}_{\text{2}}\text{O}\text{ }\text{→}\text{ }\text{2K}\text{ }\text{+}\text{ }\text{CO }\text{ }\text{ (S10)}$$

called chemical activation, is responsible for generating the pore network; (b) the formation of H_2_O ($\text{2}\text{KOH}\text{ → }\text{K}_{\text{2}}\text{O + }\text{H}_{\text{2}}\text{O}$) and CO_2_ ($\text{CO + }\text{H}_{\text{2}}\text{O → }\text{H}_{\text{2}}\text{ + C}\text{O}_{\text{2}}$ and $\text{K}_{\text{2}}\text{CO}_{\text{3}}\text{ → }\text{K}_{\text{2}}\text{O + C}\text{O}_{\text{2}}$) in the activation system positively contributes to the further development of the porosity through the gasification of carbon as shown:

$$\text{C + }\text{H}_{\text{2}}\text{O → }\text{H}_{\text{2}}\text{ + CO }\text{ }\text{ (S}\text{11}\text{)}$$

$$\text{C + C}\text{O}_{\text{2}}\text{ → }\text{2}\text{CO}\text{ }\text{(S}\text{12}\text{)}$$

namely physical activation; (c) the as-prepared metallic K (Eqs. (8-10)), efficiently intercalating into the carbon lattices of the carbon matrix during the activation, results in the expansion of the carbon lattices [S11]. After the removal of the intercalated metallic K and other K compounds by washing, the expanded carbon lattices cannot return to their previous nonporous structure and thus the high microporosity.

The development of large specific surface area and high porosity in KOH-activated carbons is the result of the synergistic, comprehensive actions including chemical activation, physical activation, and carbon lattice expansion by the metallic K intercalation. In our research, this activation mechanism is also applicable to the etching process of carbon cloth. After soaking in 1 M KOH solution and annealing at a suitable time at 600 ℃, microporous structures can be etched on the surface of carbon cloth fibers (Fig. S20), which will further increase the specific surface area of carbon cloth. When the carbon cloth is annealed at 600 ℃ for 5 h without KOH soaking, a small number of micropores will appear on the surface of the carbon cloth fiber (Fig. S21), which may be caused by gasification of carbon.

Supplementary Figures and Tables


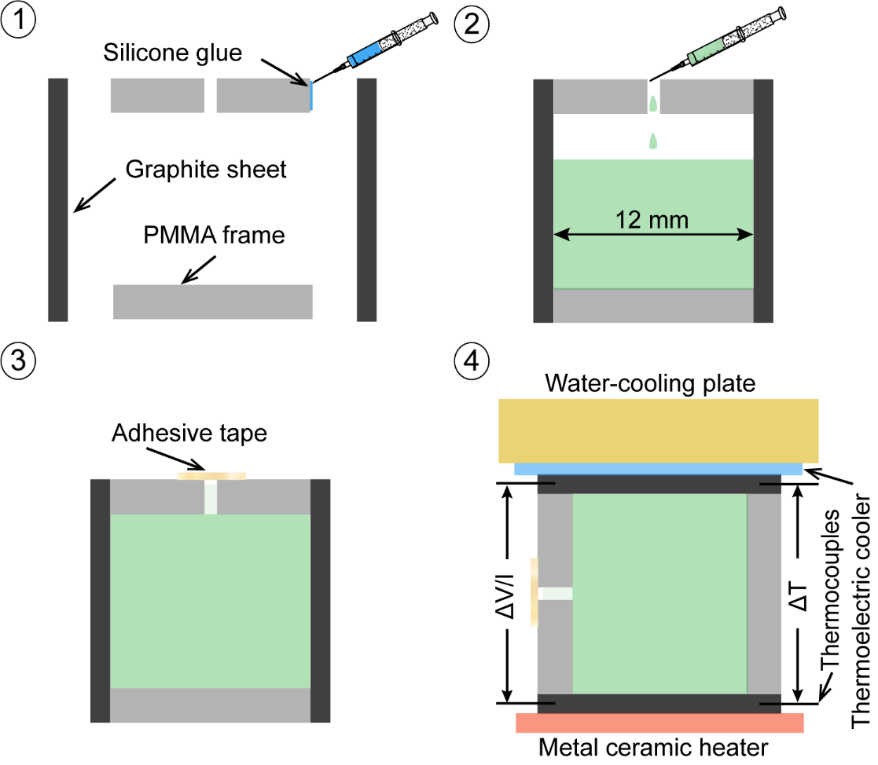


**Fig. S1** Schematic sectional drawing illustrating the assembly process of the LTC device in four steps: (1) Preparing all parts and assembling the cell frame; (2) Injecting the electrolyte to fill the cell; (3) Sealing the cell using nano transparent double-sided tape; (4) Horizontally placing the cell for testing. The temperature difference in the LTC is achieved by positioning a heater on one side and a thermoelectric cooler with a water-cooled plate on the opposite side. The water-cooled plate effectively cools the thermoelectric cooler through water circulation, helping maintain the cold side of the LTC at 20 °C. The cross-sectional area of the cell is 3.24 cm^2^, with an electrode separation of 1.2 cm.


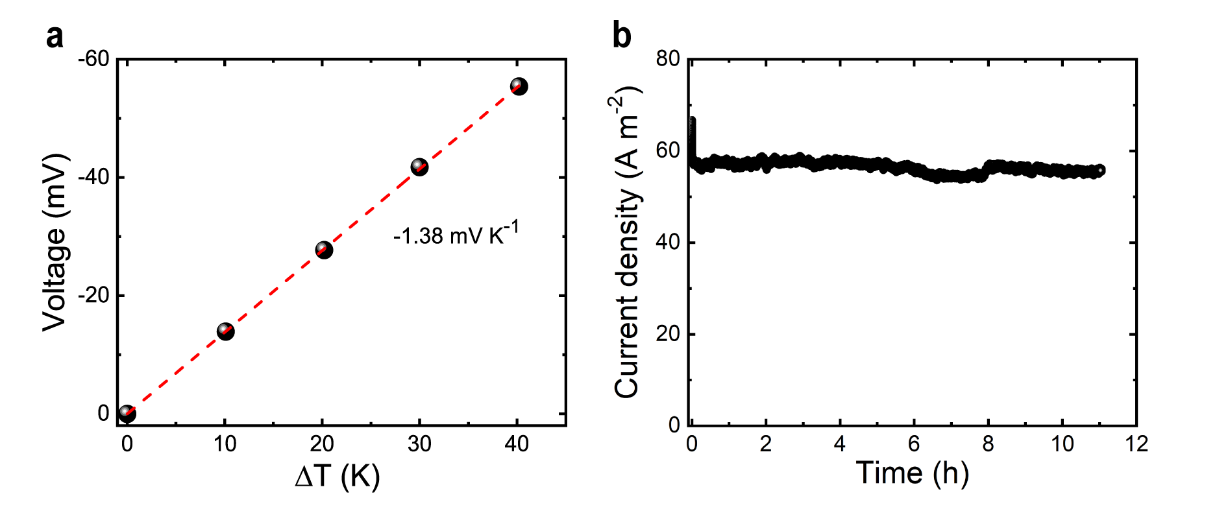


**Fig. S2** **a** The open-circuit voltage of the 0.4 M Fe(CN)_6_^3‒^/Fe(CN)_6_^4‒^ LTC across a temperature difference (ΔT) ranging from 0 to 40 K. The 0.4 M Fe(CN)_6_^3‒^/Fe(CN)_6_^4‒^ system, with a thermopower *S*_e_ of approximately ‒1.4 mV K^–1[12]^, serves as a benchmark for thermocells. To verify the accuracy of our experimental device, we tested the 0.4 M Fe(CN)_6_^3‒^/Fe(CN)_6_^4‒^ system using our experimental setup. Within the temperature range of 0 to 40 K, the measured *S*_e_ was ‒1.38 mV K^–1^, confirming the reliability of our testing apparatus. **b** Short-circuit current density (*J*_sc_) of 0.4 M Fe(CN)_6_^3‒^/Fe(CN)_6_^4‒^ LTC versus time over about 12 h short-circuit operation at a ΔT = 40 K


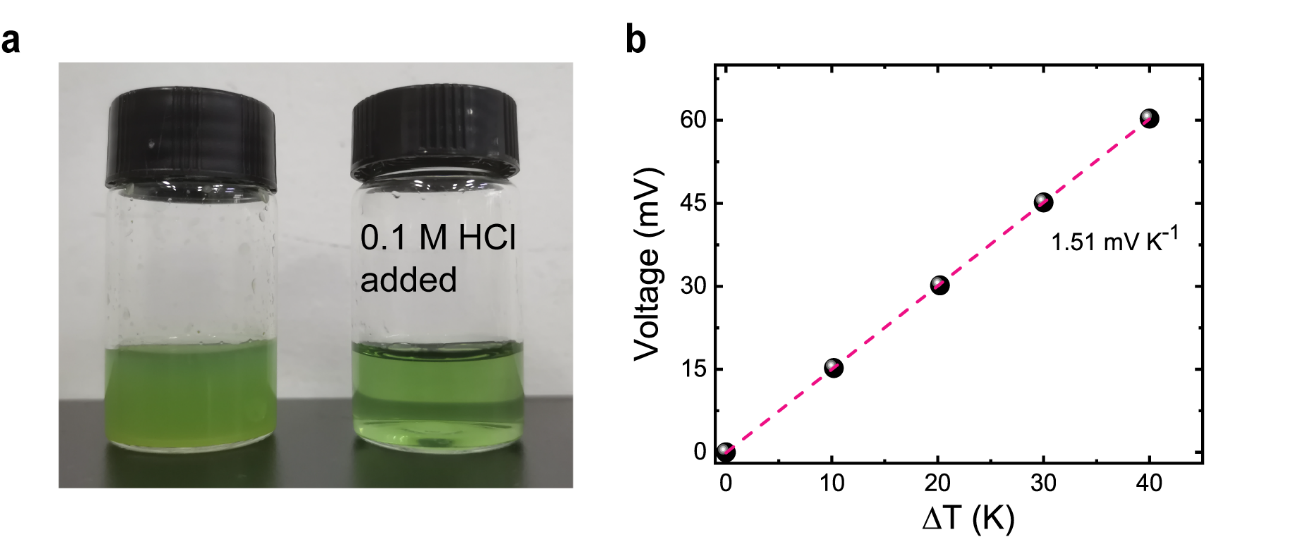


**Fig. S3 a** Photographs of electrolytes comprising 0.15 M CuCl/CuCl_2_ in 0.9 M NH_4_Cl solution with and without the addition 0.1 M HCl. The results indicate that the addition of a small amount of HCl inhibits hydrolysis and enhances dissolution. **b** Open-circuit voltage of the LTC device as a function of temperature difference (ΔT) from 0 to 40 K


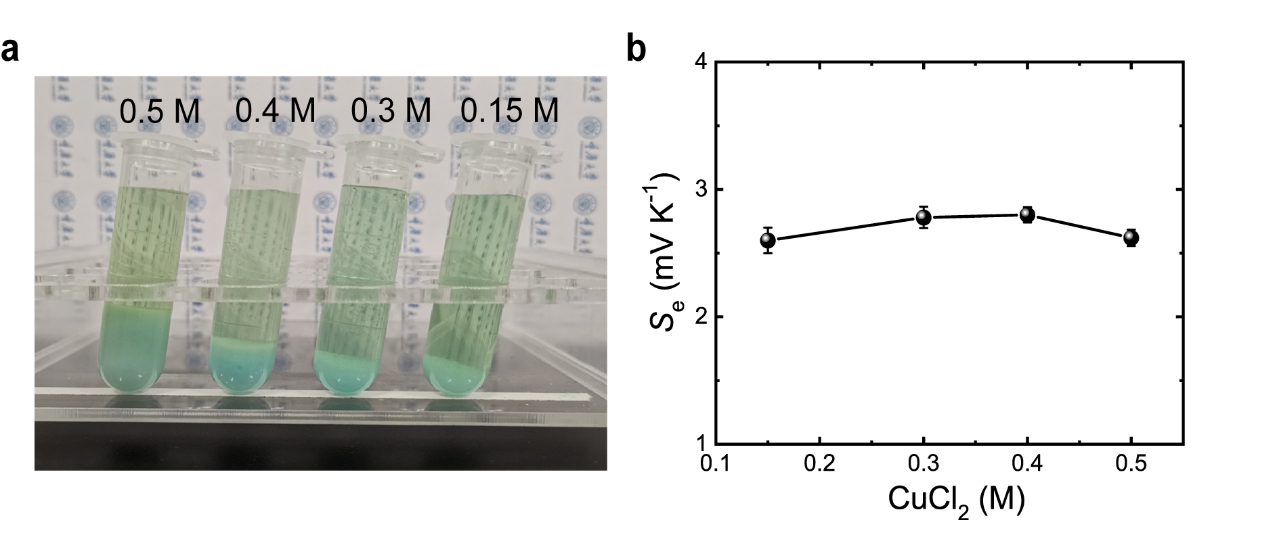


**Fig. S4 a** Photographs of electrolytes including 0.15 M CuCl and different amount of CuCl_2_ in 0.9 M NH_4_Cl and 0.1 M HCl solutions with 3 M (NH_4_)_2_SO_4_ added. An increase in the CuCl_2_ content corresponds to a greater amount of precipitate produced. **b** The corresponding *S*_e_ as a funciton of CuCl_2_ concentration. The *S*_e_ reaches its maximum value when the CuCl_2_ concentration is 0.4 M.


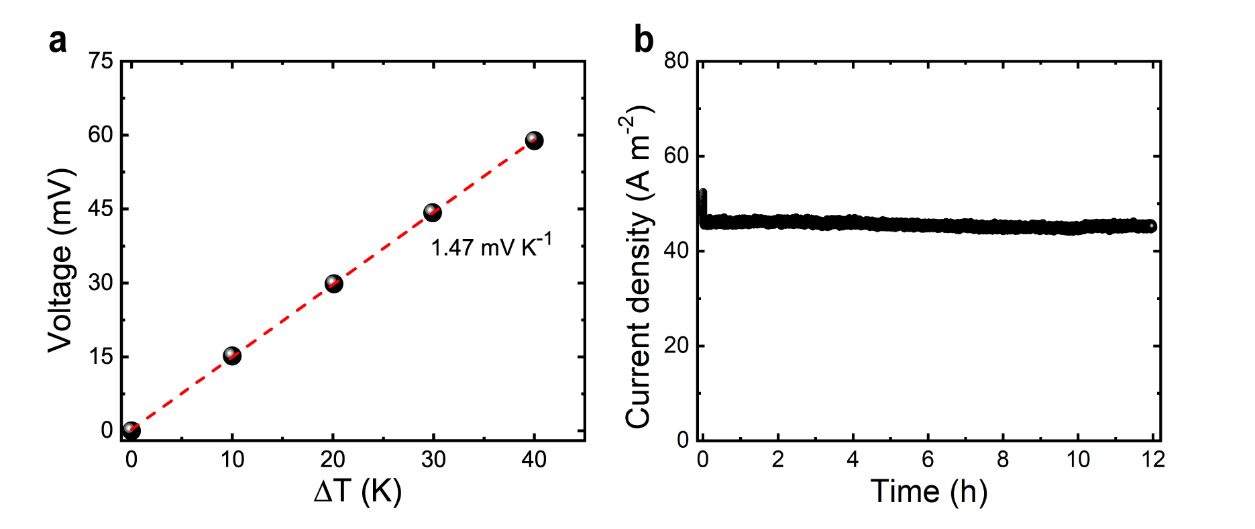


**Fig. S5** Thermogalvanic properties of the selected pristine electrolyte for the LTC. The pristine electrolyte comprises 0.15 M CuCl, 0.4 M CuCl_2_, 0.9 M NH_4_Cl, and 0.1 M HCl. **a** Open-circuit voltage of this LTC as a function of ΔT ranging from 0 to 40 K. **b** Short-circuit current density (*J*_sc_) versus time over a 12 h short-circuit operation. The *J*_sc_ do not fluctuate, indicating excellent stability of the LTC.


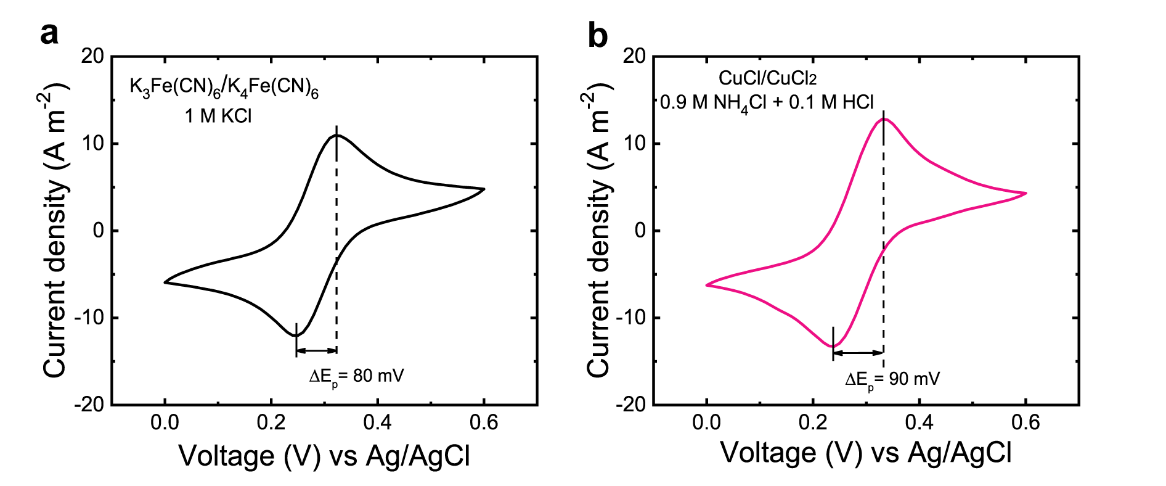


**Fig. S6** Cyclic voltammetry (CV) curves scanned at 10 mV s^-1^ for (**a**) 2 mM K_3_Fe(CN)_6_/K_4_Fe(CN)_6_ in 1 M KCl aqueous solution and (**b**) 2 mM CuCl/CuCl_2_ in 0.9 M NH_4_Cl + 0.1 M HCl aqueous solution. Both the working electrode and the opposing electrode are graphite electrodes, and reference electrode is Ag/AgCl electrode.


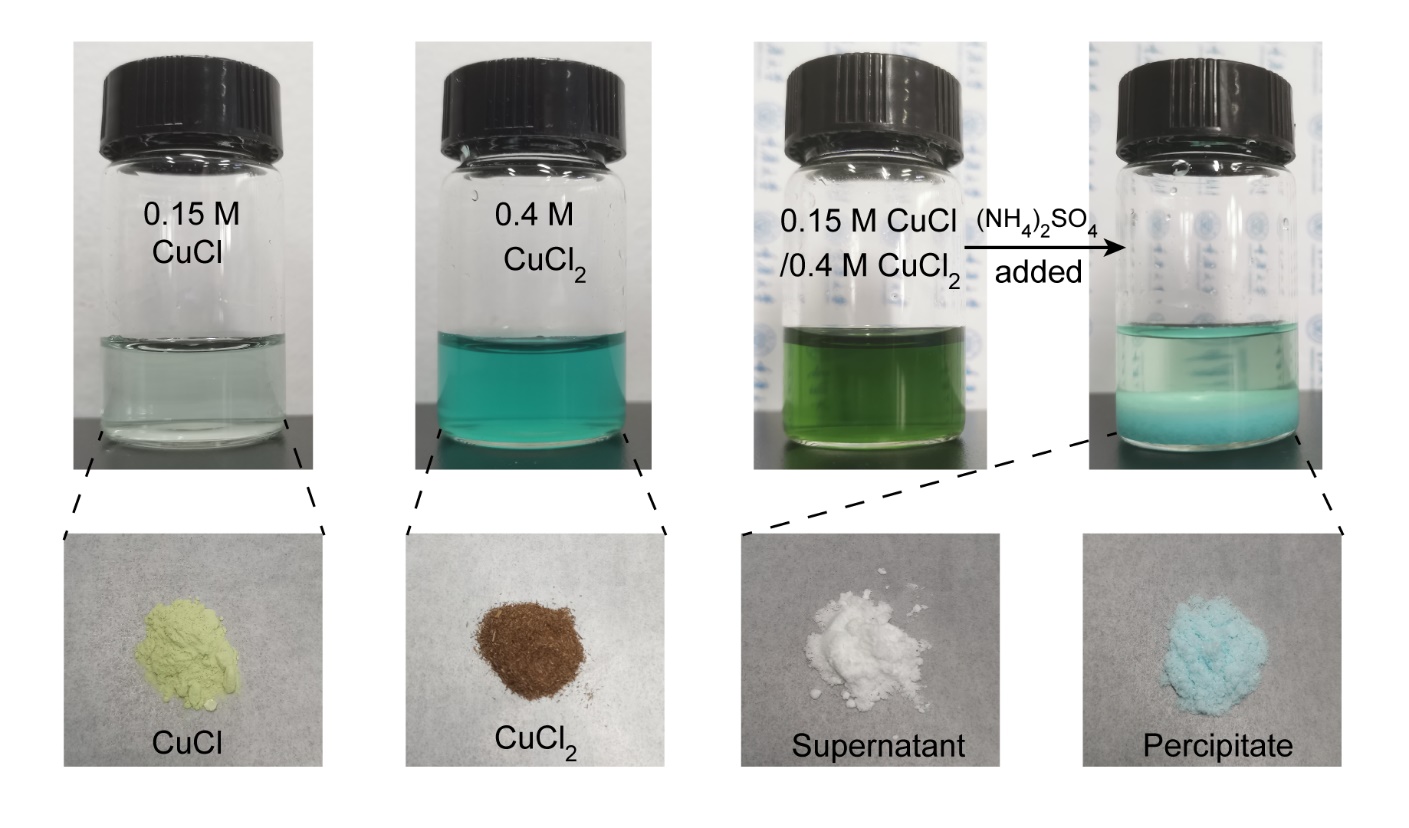


**Fig. S7** Photographs of the electrolytes (top row), left to right: 0.15 M CuCl, 0.4 M CuCl_2_, 0.15 M CuCl/0.4 M CuCl_2_ and 0.15 M CuCl/0.4 M CuCl_2_ + 4 M (NH_4_)_2_SO_4_ in 0.9 M NH_4_Cl solution with 0.1 M HCl. The bottom row shows the corresponding dried powders obtained through vacuum drying. CuCl appears as a white powder; however, upon exposure to moisture in the air, it slightly oxidizes and takes on a light yellow color. The dried powders from the supernatant are white, indicating a very low content of Cu^2+^ ions.


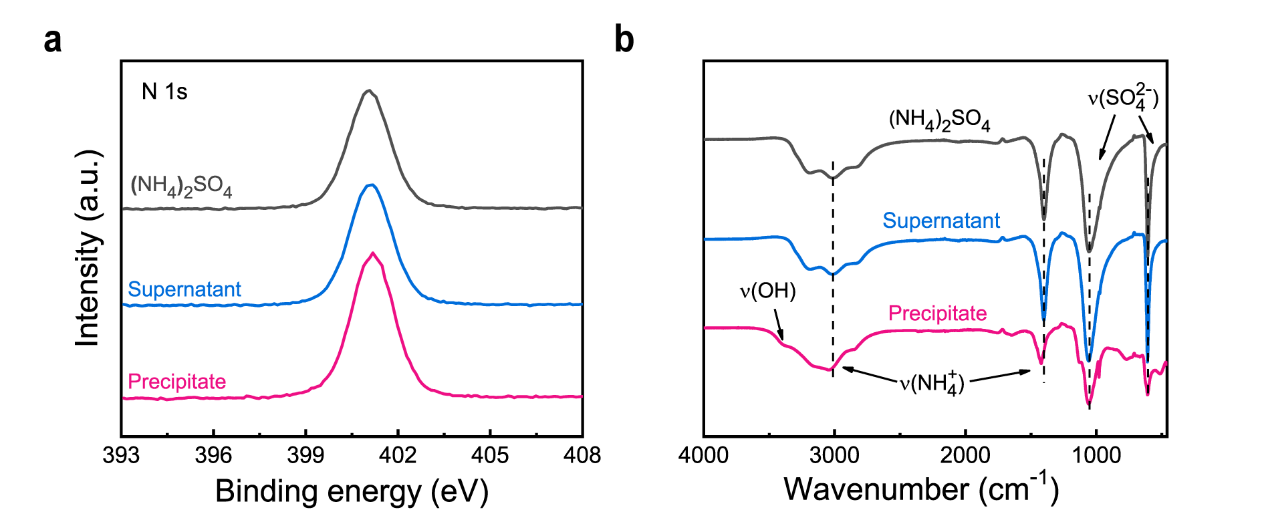


**Fig. S8** **a** N 1s XPS spectra for pure (NH_4_)_2_SO_4_ and the dried powders from the supernatant and precipitate in the (NH_4_)_2_SO_4_ added electrolyte. **b** FTIR spectra for pure (NH_4_)_2_SO_4_ and dried powders from the supernatant and precipitate in the (NH_4_)_2_SO_4_ added electrolyte


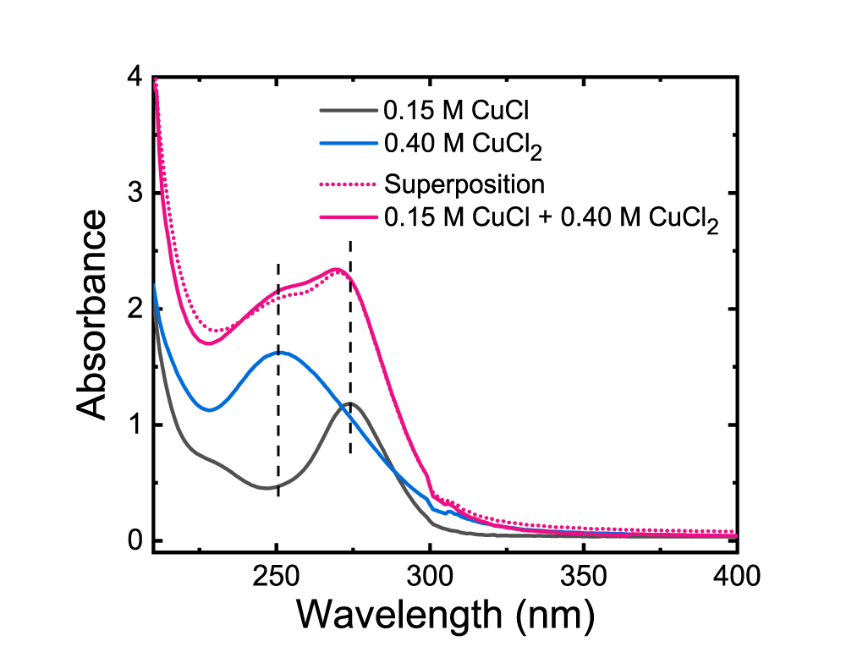


**Fig. S9** UV-vis absorption spectra of Cu^+^ or/and Cu^2+^ in different aqueous solutions. The dashed line represents the superposition of the UV-Vis absorption spectra under 0.15 M CuCl and 0.4 M CuCl_2_ conditions. It is evident that the dashed line closely matches the UV-Vis absorption spectrum of the solution containing 0.15 M CuCl + 0.4 M CuCl_2_ at the absorption band. This observation suggests that the shift of the Cu^+^ absorption peak in the presence of Cu^2+^ is attributable to the superposition of the Cu^2+^ absorption band.


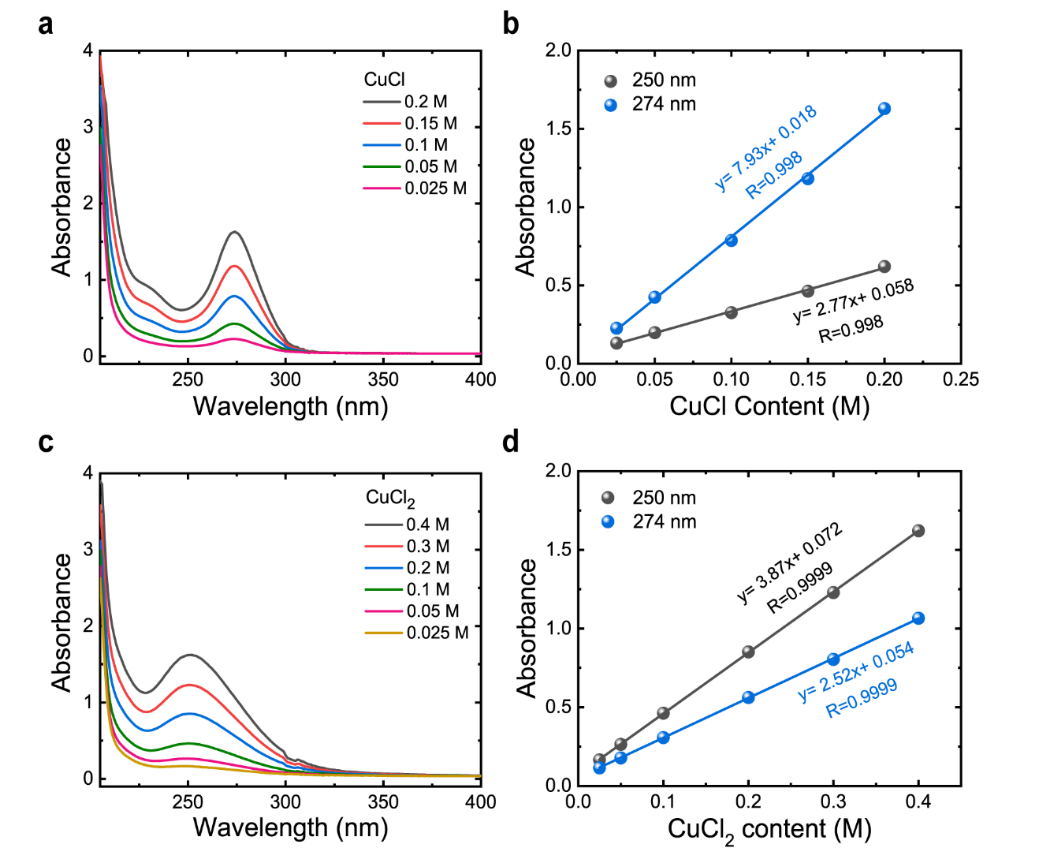


**Fig. S10** Cuprous chloride and copper chloride scale measurement. **a** UV-vis absorption spectra for CuCl solutions with a gradient of concentration. **b** Linear fit between CuCl content and absorbance at 250 nm and 274 nm. **c** UV-vis absorption spectra for CuCl_2_ solution with a gradient of concentration. **d** Linear fit between CuCl_2_ content and absorbance at 250 nm and 274 nm


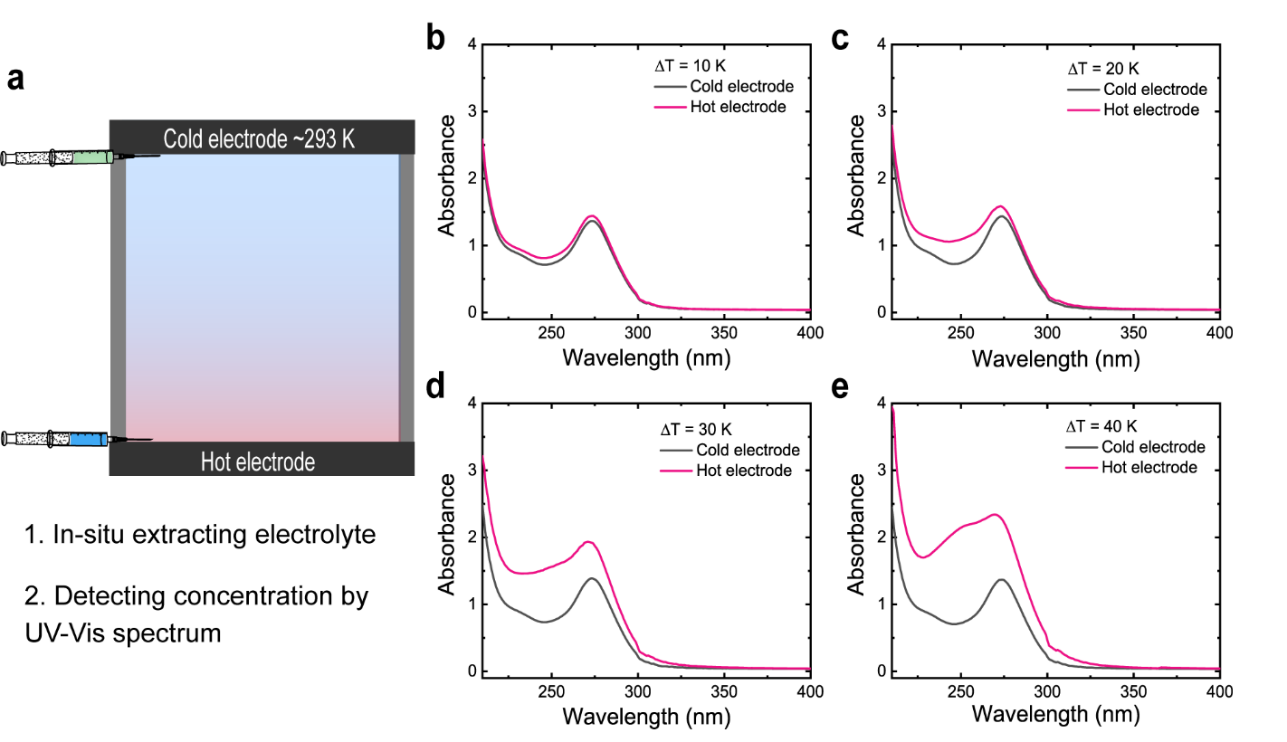


**Fig. S11** **a** Schematic representation of the in-situ method for measuring redox species concentration in TC-LTC using a solution containing 0.15 M CuCl/0.4 M CuCl_2_, 0.1 M HCl, 0.9 M NH_4_Cl, and 4 M (NH_4_)_2_SO_4_. (**b**-**e**) UV-Vis spectra of solution at cold and hot electrode in TC-LTC under different temperature difference. The extracted solution samples were diluted ~ 350 times for UV-Vis analysis. The temperature at the cold electrode is controlled at about 293 K (20 ℃).


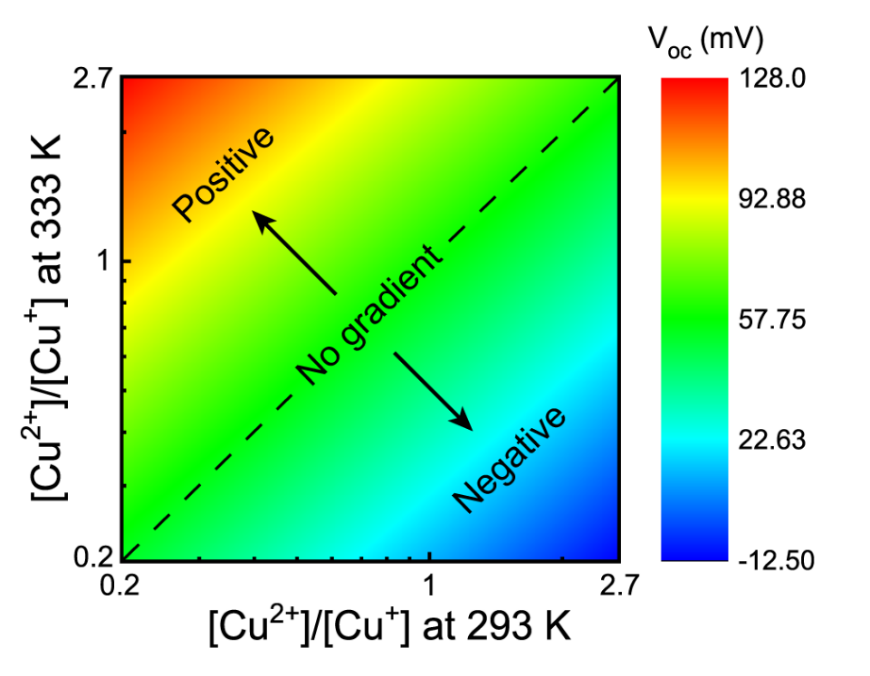


**Fig. S12** Simulated V_oc_ of the TC-LTC depending on the concentration ratio of [Cu^2+^]/[Cu^+^] at the two electrodes. The temperatures at the cold and hot electrodes were 293 and 333 K, respectively. This simulation is based on Eq. (S6) in Note S1


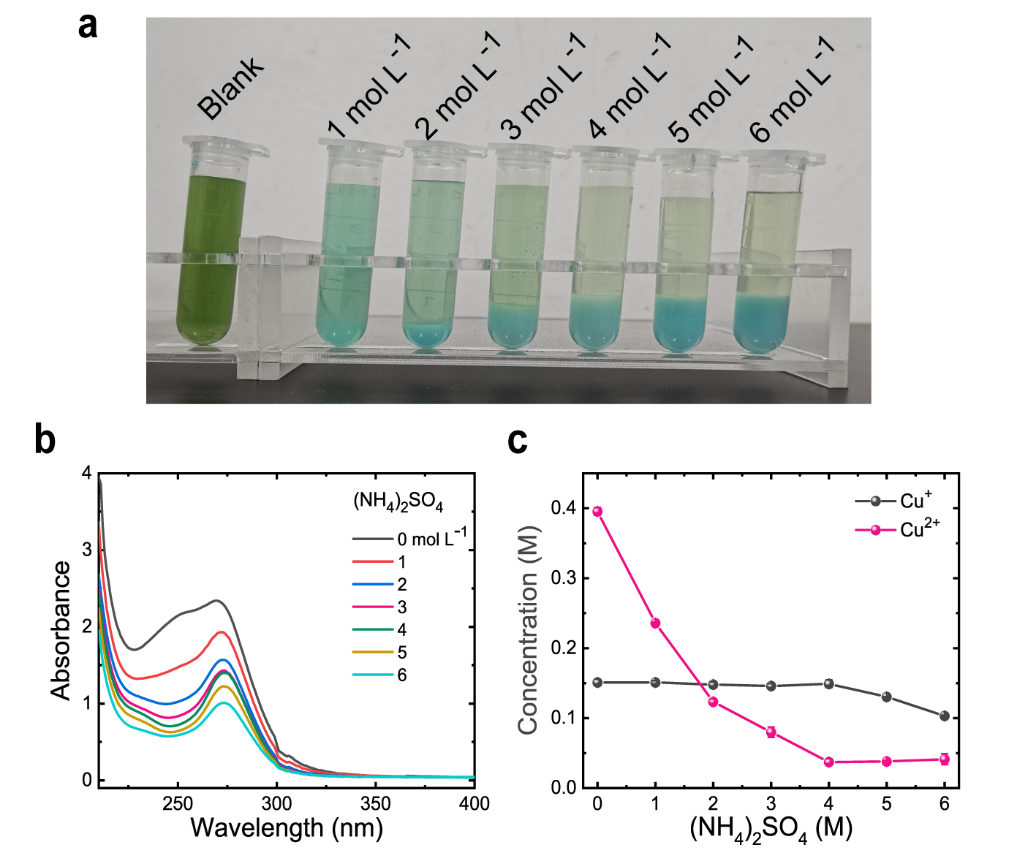


**Fig. S13** **a** Photographs of the 0.15 M CuCl/0.4 M CuCl_2_ electrolyte with different amount of (NH_4_)_2_SO_4_. **b** UV-Vis spectra of CuCl and CuCl_2_ in electrolyte with different amount of (NH_4_)_2_SO_4_. **c** Concentrations of Cu^+^ and Cu^2+^ in electrolyte with different amount of (NH_4_)_2_SO_4_. These concentrations are calculated from the corresponding UV-Vis spectra. When the concentration of (NH_4_)_2_SO_4_ exceeded 4 M, the Cu^+^ content began to decrease, suggesting that the increase in the precipitation content after the addition of more than 4 M (NH_4_)_2_SO_4_ came from concomitant Cu⁺ crystallization.


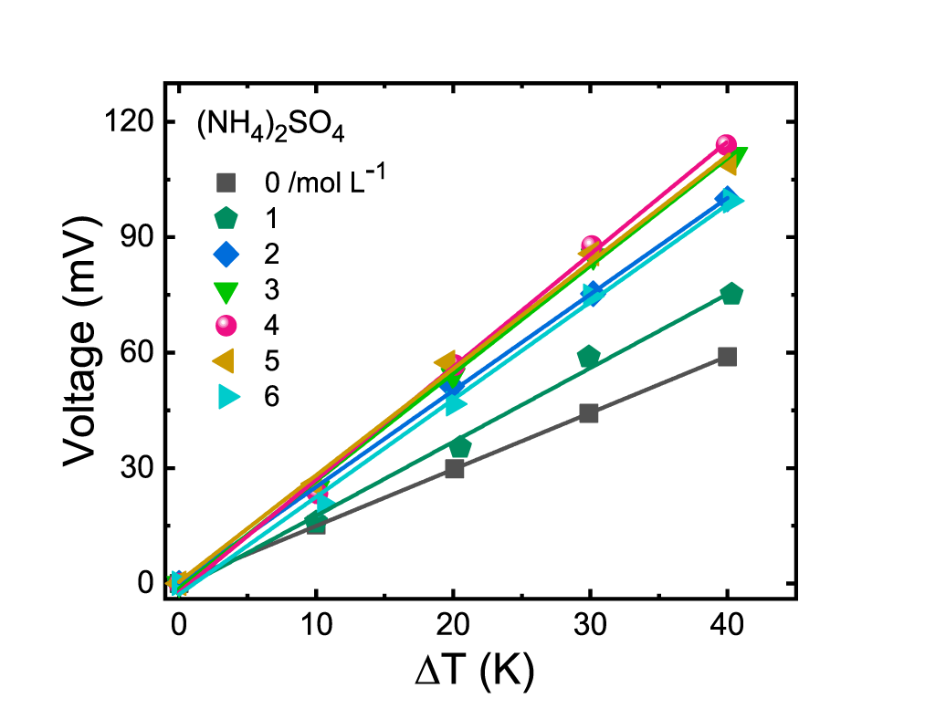


**Fig. S14** Plot of measured V_oc_ versus ΔT for the TC-LTC with different amounts of added (NH_4_)_2_SO_4_


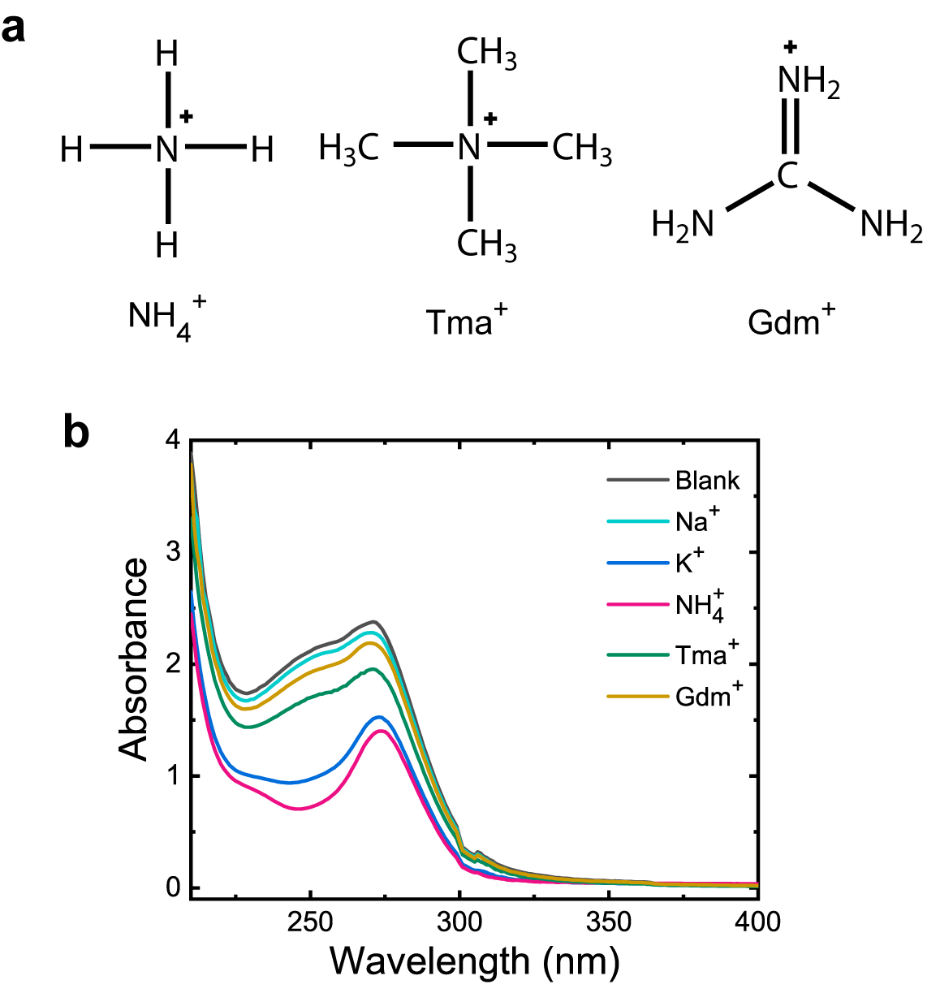


**Fig. S15** **a** Chemical structures of ammonium (NH_4_^+^), tetramethylammonium (Tma^+^), and guanidinium (Gdm^+^). **b** UV-Vis spectra of the electrolyte containing different cation additives


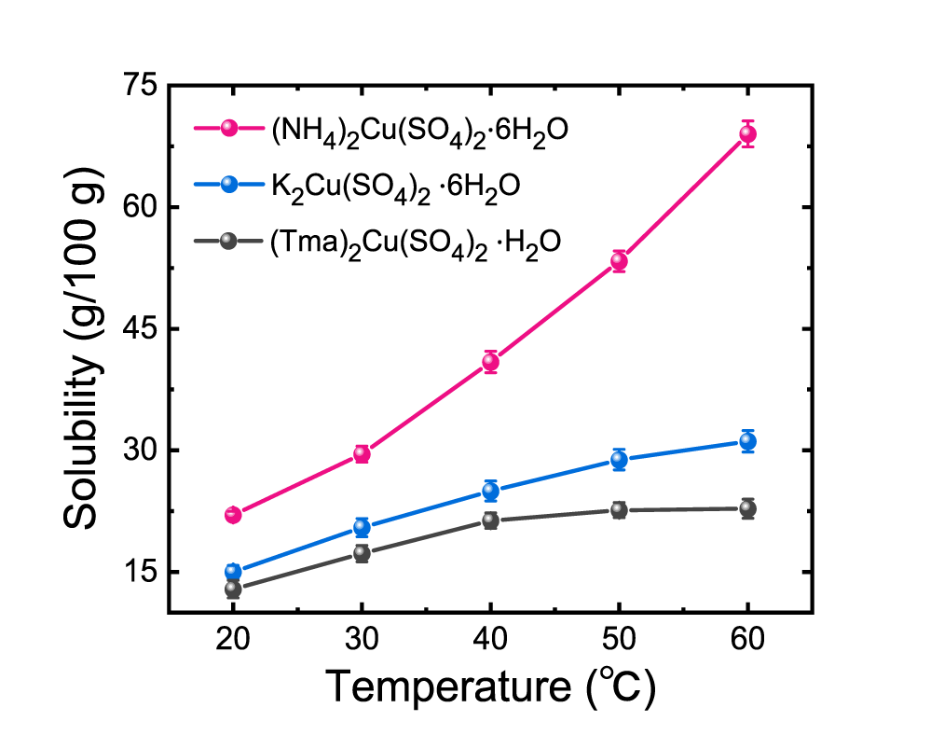


**Fig. S16** Plot of measured solubility versus temperature for (NH_4_)_2_Cu(SO_4_)_2_∙6H_2_O, K_2_Cu(SO_4_)_2_∙6H_2_O, and (Tma)_2_Cu(SO_4_)_2_∙H_2_O


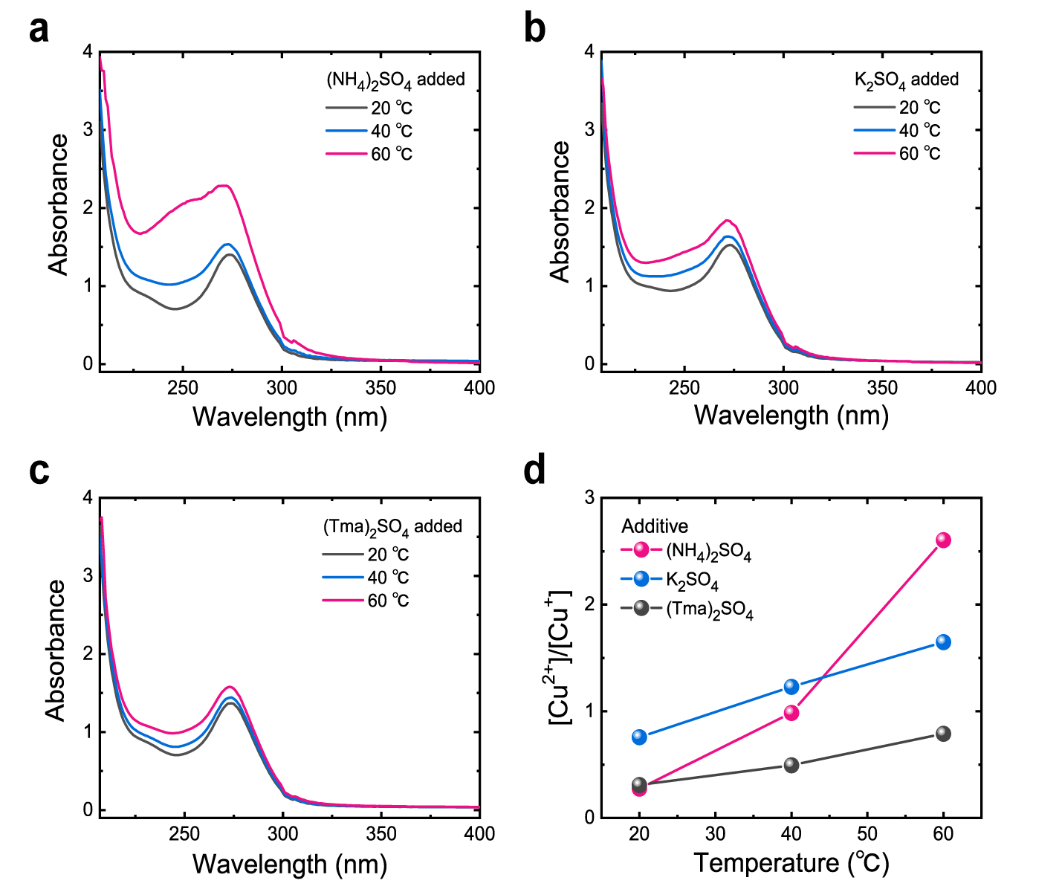


**Fig. S17** UV-Vis spectra at increasing temperatures following the addition of **a** (NH_4_)_2_SO_4_, **b** K_2_SO_4_, and **c** (Tma)_2_SO_4_. **d** Plot of the concentration ratio of [Cu^2+^]/[Cu^+^] versus temperature for the electrolyte with various additive, which was calculated from the corresponding UV-Vis spectrum by the Lambert-Beer law


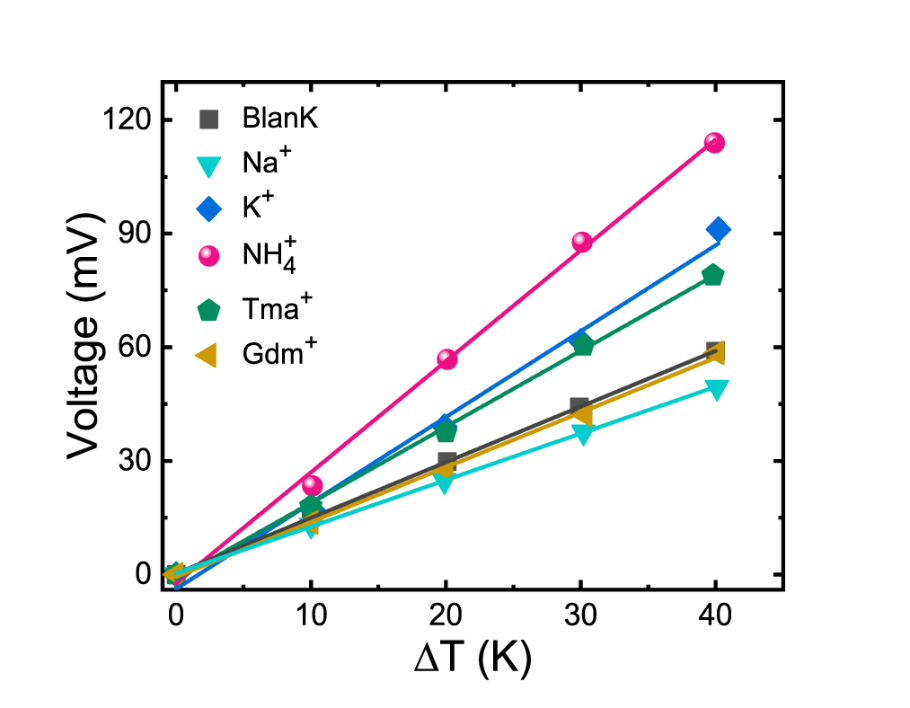


**Fig. S18** Plot of measured V_oc_ versus ΔT for the TC-LTC with different cation additives


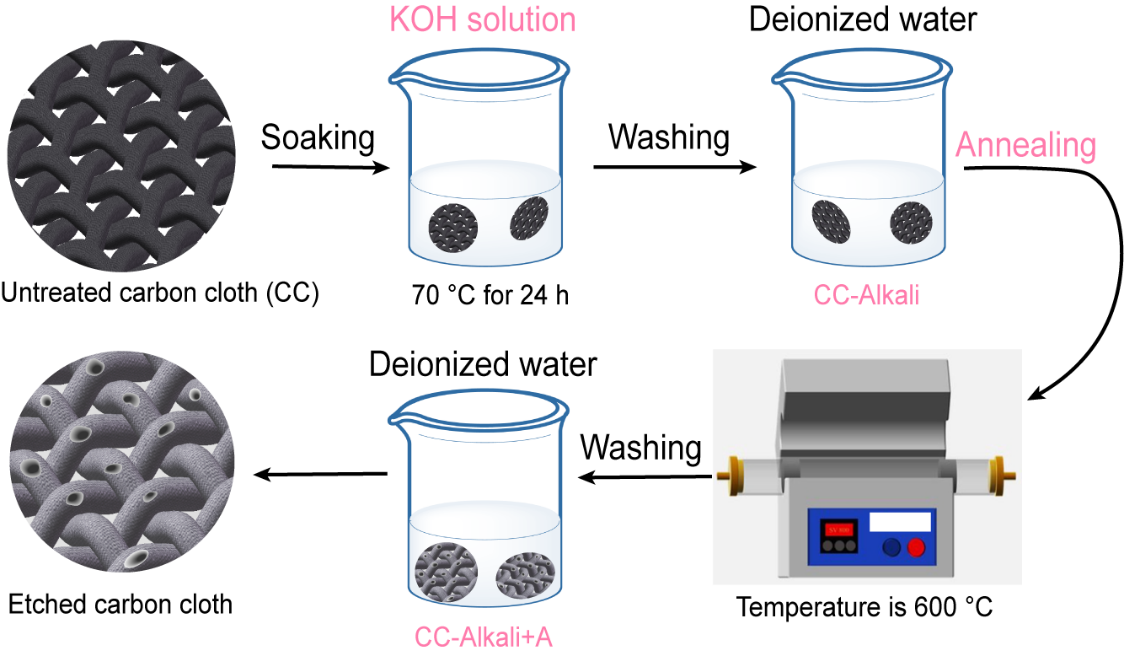


**Fig. S19** Diagram of the carbon cloth etching process. The cut carbon cloth is immersed in a 1 M KOH solution for 24 hours, concurrently heated at 70 °C. After immersion, the carbon cloth is removed and rinsed three times with deionized water to eliminate excess KOH. The etched carbon cloth is then obtained by annealing at 600 °C for varying durations in a tube furnace and subsequently cleaned


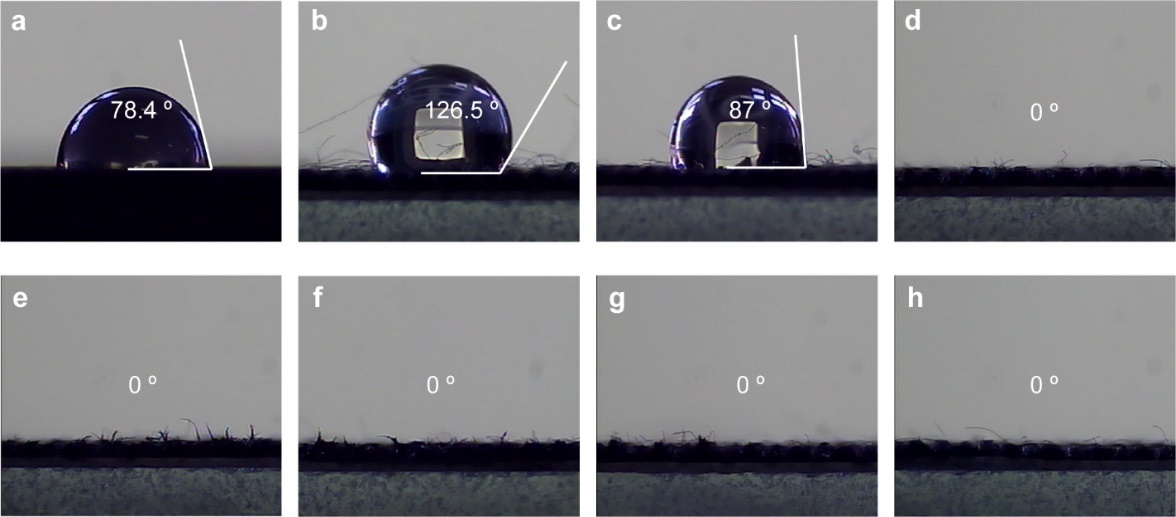


**Fig. S20** Photographs of water drops on three different electrodes. **a** Graphite sheet; **b** untreated carbon cloth; **c** treated carbon cloth soaked in 1 M KOH; **d** carbon cloth annealed for 5 h without soaking in 1 M KOH; and carbon cloth annealed at 600 ℃ for **e** 1 h, **f** 3 h, **g** 5 h, and **h** 7 h after soaking in 1 M KOH


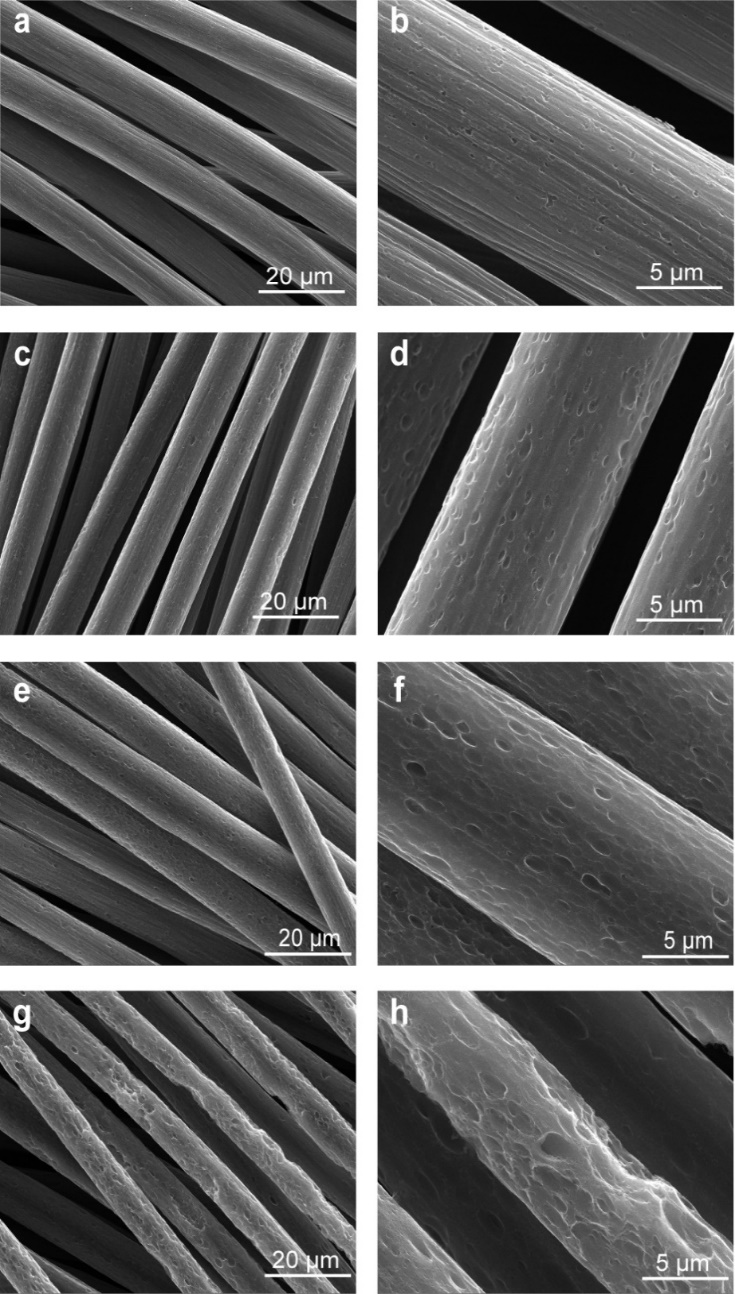


**Fig. S21** SEM images of the surface of carbon cloth electrodes with increasing annealing times, 1 h (**a**, **b**), 3 h (**c**, **d**), 5 h (**e**, **f**), 7 h (**g**, **h**)


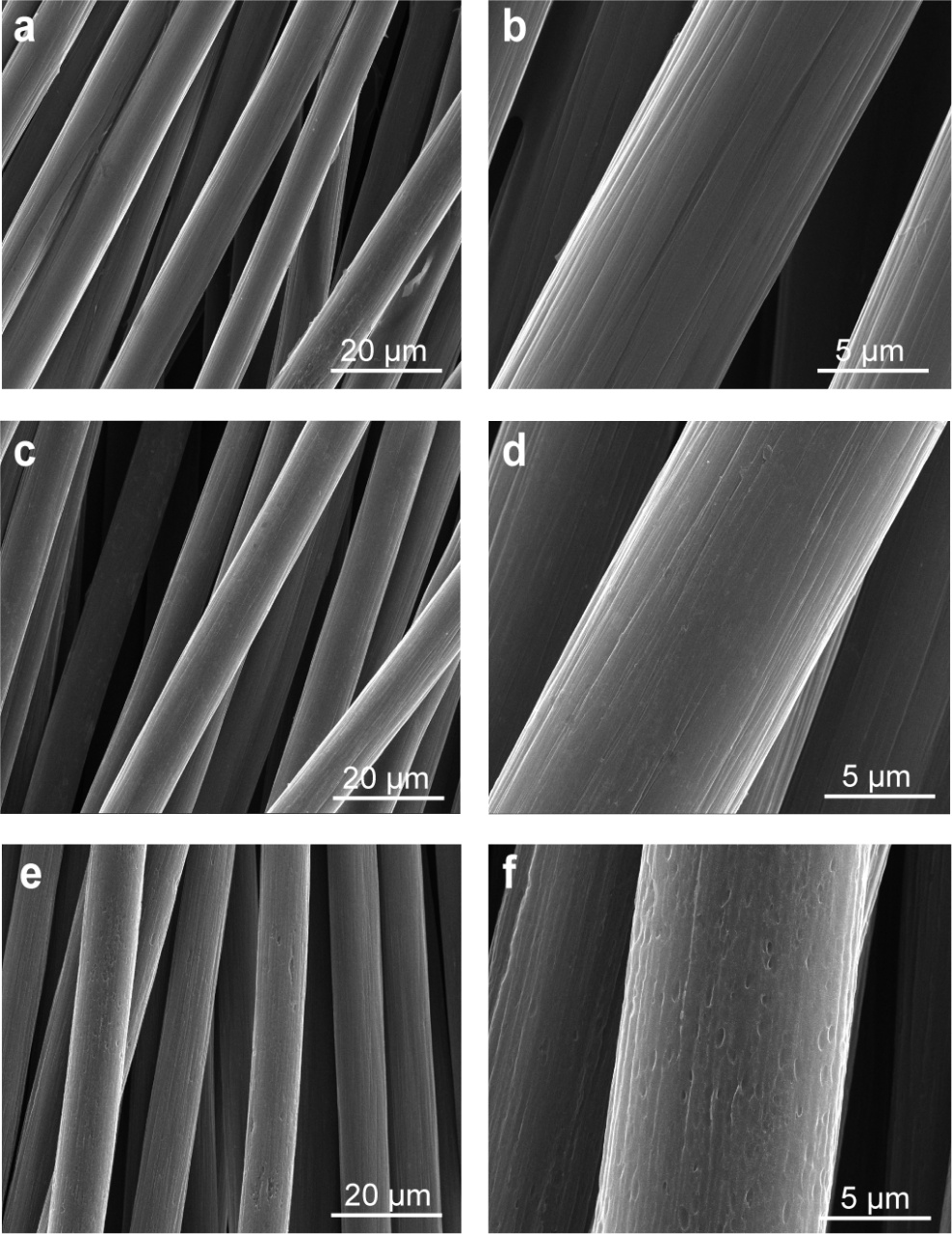


**Fig. S22** SEM images of the surface of (**a**, **b**) untreated carbon cloth, (**c**, **d**) alkalized carbon cloth, and (**e**, **f**) carbon cloth annealed at 600 ℃ for 5 h without KOH immersion


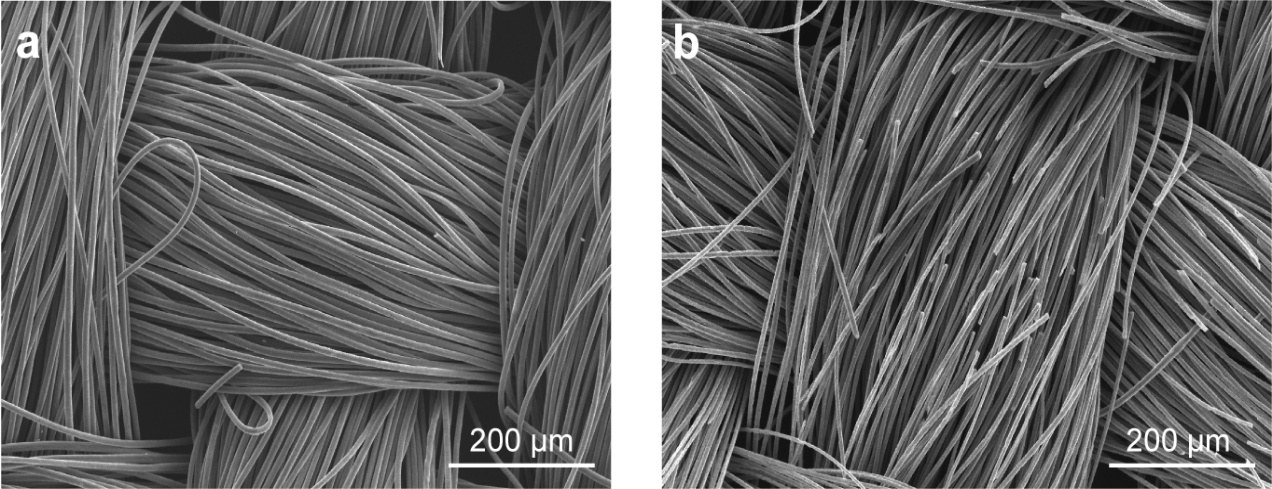


**Fig. S23** SEM images of the surface of carbon cloth annealed at 600 ℃ for **a** 5 h and **b** 7 h


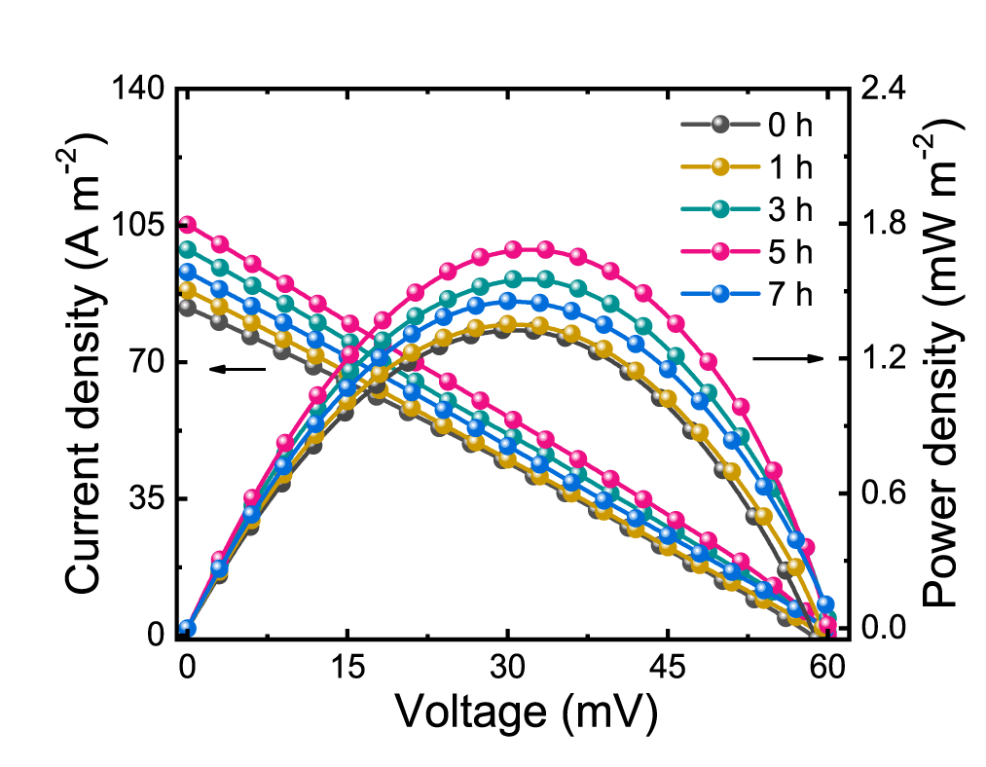


**Fig. S24** Current-voltage and power-voltage curves for LTC with carbon cloth electrodes annealed at 600 ℃ for different times


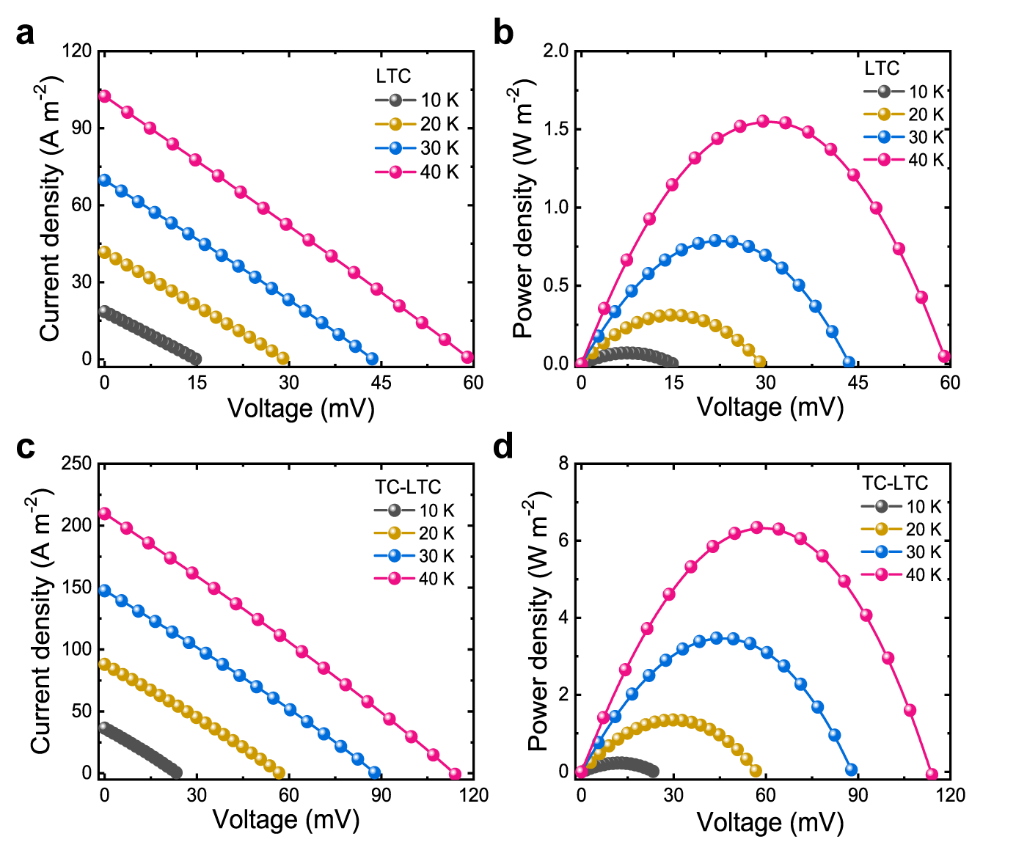


**Fig. S25** (**a**, **c**) Current-voltage curves for the LTC and TC-LTC at different values of ΔT, and their corresponding power densities (**b**, **d**) with CC-Alkali+A

**Table S1** Summary of recently reported intrinsic n-type TECs

| Redox  couple | Electrolyte | Electrode | | Se  (mV K^-1^) | P_max_ (ΔT)^-2^  (mW m^-2^ K^-2^) | Refs. |
| --- | --- | --- | --- | --- | --- | --- |
| $\text{Fe(CN)}_{\text{6}}^{\text{3-/4-}}$ | 0.4 M $\text{Fe(CN)}_{\text{6}}^{\text{3-/4-}}$ | Pt | | 1.44 | 0.592 | [S13] |
|  | 0.4 M $\text{Fe(CN)}_{\text{6}}^{\text{3-/4-}}$ | Activated carbon cloth | | 1.40 | 1.80 | [S14] |
|  | 0.4 M $\text{Fe(CN)}_{\text{6}}^{\text{3-/4-}}$ in methanol/water | SWNT | | 2.90 | 0.64 | [S15] |
|  | 0.4 M $\text{Fe(CN)}_{\text{6}}^{\text{3-/4-}}$with guanidinium/ urea added | Graphite | | 4.2 | 1.1 | [S1] |
|  | 0.4 M $\text{Fe(CN)}_{\text{6}}^{\text{3-/4-}}$with guanidinium added | Carbon fiber | | 3.73 | 7.08 | [S3] |
| Fe^2+/3+^ | 0.5 M Fe^2+/3+^-Cl | Pt | | 1.04 | 0.256 | [S13] |
|  | 0.2 M Fe^2+/3+^-NO_3_ + 1 M H NO_3_ | Au | | 1.38 | 0.163 | [S16] |
|  | 0.8 M Fe^2+/3+^-ClO_4_ | DWCNT sheet | | 1.65 | 0.41 | [S17] |
|  | 0.8 M Fe^2+/3+^-ClO_4_ + 1 M HClO_4_ | Carbon cloth | | 1.50 | 1.19 | [S18] |
|  | 0.8 M Fe^2+/3+^-ClO_4_ | Fe-N-C coated carbon cloth | | 1.64 | 1.92 | [S19] |
| I^‒^/I_3_^‒^ | 5 mM I^‒^/I_3_^‒^ with methylcellulose | Graphite | | 9.62 | 0.36 | [S20] |
|  | 0.4 M I^‒^/I_3_^‒^ with CsCl added | Graphite | | 1.2 | 0.296 | [S21] |
|  | 2.5 mM KI_3_, 10 mM KI, 0.2 M KCl | Pt wire mesh | | 1.97 | 0.0147 | [S22] |
| Cu/Cu^2+^ | 0.7 M CuSO_4_ + 0.1 M H_2_SO_4_ | Cu foil | | 0.70 | 0.0012 | [S23] |
|  | 0.7 M CuSO_4_ + 0.1 M H_2_SO_4_ with ethylenediamine and (NH_4_)_2_SO_4_ added | Cu foil | | 2.12 | 0.676 | [S24] |
|  | 1.2 M CuSO_4_ + 0.1 M H_2_SO_4_ with (NH_4_)_2_SO_4_ added | 3D multi-structured Cu | | 1.66 | 0.71 | [S4] |
| $\text{Co(bpy)}_{\text{3}}^{\text{2+/3+}}$ | 0.025 M Co(bpy)_3_^2+/3+^ in [EMIM][NTf_2_] | Pt | | 1.60 | 0.028 | [S25] |
|  | 0.05 M Co(SAR)(OTf)_4/5_ in DMSO with HOTf | Pt | | 2.04 | 0.011 | [S26] |
|  | 0.1 M Co(bpy)_3_(NTf_2_)_2/3_ in MPN | Pt | | 1.92 | 0.102 | [S27] |
| CuCl/CuCl_2_ | 0.15MCuCl/0.4M CuCl_2_ in 0.1 M HCl and 0.9 M LiCl with (NH_4_)_2_SO_4_ added | Graphite | 2.93 | | 3.968 | This work |

**Table S2** Summary of data of various thermoelectric materials, reported in the literatures and this work, used for CPM estimation

| Type | Material | Temperature  (K) | Material CPM  ($ W−1) | Reference |
| --- | --- | --- | --- | --- |
| LTCs | 0.15MCuCl/0.4M CuCl_2_ in 0.1 M HCl and 0.9 M LiCl with (NH_4_)_2_SO_4_ added | 333 | 1.17 | This work |
|  | K_3_Fe(CN)_6_/K_4_Fe(CN)_6_/GdmCl solution | 318 | 1.56 | [S3] |
| OTECs | PEDOT | RT | 116.96 | [S28] |
|  | CNTs | 310 | 133.57 | [S29] |
| ITECs | Bi_2_Te_3_ | 333 | 1979.18 | [S30] |
|  | Mg_3.2_Bi_1.498_Sb_0.5_Te_0.002_ | 325 | 53.79 | [S31] |
|  | PbTe | 380 | ~2155.31 | [S32] |
|  | SnSe | 320 | ~336.55 | [S33] |

Material CPM: the Cost-Performance Metric (CPM) is simply calculated from the maximum device performance (P_max_) and the corresponding raw material cost (C_m_) by: CPM=C_m_/P_max_

**Supplementary Movie S1** TC-LTC module to power electronic devices at ΔT = 40 K

Supplementary References

1. J. Duan, G. Feng, B. Yu, J. Li, M. Chen et al., Aqueous thermogalvanic cells with a high Seebeck coefficient for low-grade heat harvest. Nat. Commun. **9**(1), 5146 (2018). <https://doi.org/10.1038/s41467-018-07625-9>
2. S. Sahami, M.J. Weaver, Entropic and enthalpic contributions to the solvent dependence of the thermodynamics of transition-metal redox couples. J. Electroanal. Chem. Interfacial Electrochem. **122**, 171–181 (1981). <https://doi.org/10.1016/S0022-0728(81)80148-9>
3. B. Yu, J. Duan, H. Cong, W. Xie, R. Liu et al., Thermosensitive crystallization-boosted liquid thermocells for low-grade heat harvesting. Science **370**(6514), 342–346 (2020). <https://doi.org/10.1126/science.abd6749>
4. B. Yu, H. Xiao, Y. Zeng, S. Liu, D. Wu et al., Cost-effective n-type thermocells enabled by thermosensitive crystallizations and 3D multi-structured electrodes. Nano Energy **93**, 106795 (2022). <https://doi.org/10.1016/j.nanoen.2021.106795>
5. D. D. Davis, K. L. Stevenson, C. R. Davis, Photooxidation of dichloro- and trichlorocuprate(Ⅰ) ions in acid solution. J. Am. Chem. Soc. **100**(17), 5344-5349 (1978). <https://doi.org/10.1021/ja00485a015>
6. A. Müllertz, Y. Perrie, T. Rades, UV/Vis spectrophotometry and UV imaging. Springer (2016). <https://doi.org/doi.org/10.1007/978-1-4939-4029-5_1>
7. D. Lozano-Castelló, J.M. Calo, D. Cazorla-Amorós, A. Linares-Solano, Carbon activation with KOH as explored by temperature programmed techniques, and the effects of hydrogen. Carbon **45**(13), 2529–2536 (2007). <https://doi.org/10.1016/j.carbon.2007.08.021>
8. J. Wang, S. Kaskel, KOH activation of carbon-based materials for energy storage. J. Mater. Chem. **22**(45), 23710 (2012). <https://doi.org/10.1039/c2jm34066f>
9. Z. Zhang, J. Xi, H. Zhou, X. Qiu, KOH etched graphite felt with improved wettability and activity for vanadium flow batteries. Electrochim. Acta **218**, 15–23 (2016). <https://doi.org/10.1016/j.electacta.2016.09.099>
10. H. Wang, Q. Gao, J. Hu, High hydrogen storage capacity of porous carbons prepared by using activated carbon. J. Am. Chem. Soc. **131**(20), 7016–7022 (2009). <https://doi.org/10.1021/ja8083225>
11. J. Romanos, M. Beckner, T. Rash, L. Firlej, B. Kuchta et al., Nanospace engineering of KOH activated carbon. Nanotechnology **23**(1), 015401 (2012). <https://doi.org/10.1088/0957-4484/23/1/015401>
12. J. Duan, B. Yu, L. Huang, B. Hu, M. Xu et al., Liquid-state thermocells: Opportunities and challenges for low-grade heat harvesting. Joule **5**(4), 768–779 (2021). <https://doi.org/10.1016/j.joule.2021.02.009>
13. K. Kim, S. Hwang, H. Lee, Unravelling ionic speciation and hydration structure of Fe(III/II) redox couples for thermoelectrochemical cells. Electrochim. Acta **335**, 135651 (2020). <https://doi.org/10.1016/j.electacta.2020.135651>
14. L. Zhang, T. Kim, N. Li, T.J. Kang, J. Chen et al., High power density electrochemical thermocells for inexpensively harvesting low-grade thermal energy. Adv. Mater. **29**(12), 1605652 (2017). <https://doi.org/10.1002/adma.201605652>
15. T. Kim, J.S. Lee, G. Lee, H. Yoon, J. Yoon et al., High thermopower of ferri/ferrocyanide redox couple in organic-water solutions. Nano Energy **31**, 160–167 (2017). <https://doi.org/10.1016/j.nanoen.2016.11.014>
16. M.A. Buckingham, F. Marken, L. Aldous, The thermoelectrochemistry of the aqueous iron(ii)/iron(iii) redox couple: significance of the anion and pH in thermogalvanic thermal-to-electrical energy conversion. Sustain. Energy Fuels **2**(12), 2717–2726 (2018). <https://doi.org/10.1039/c8se00416a>
17. L. Hwan, Y. Jung, K. Hyeon, Y. Jae, K. June, Stacked double-walled carbon nanotube sheet electrodes for electrochemically harvesting thermal energy. Carbon **147**, 559–565 (2019). <https://doi.org/10.1016/j.carbon.2019.03.033>
18. W. Li, C. Gao, J. Ma, J. Qiu, S. Wang, Simultaneous enhancement of thermopower and ionic conductivity for N-type Fe(III/II) thermocell. Mater. Today Energy **30**, 101147 (2022). <https://doi.org/10.1016/j.mtener.2022.101147>
19. S.-M. Jung, S.-Y. Kang, B.-J. Lee, J. Lee, J. Kwon et al., Fe─N─C electrocatalyst for enhancing Fe(II)/Fe(III) redox kinetics in thermo-electrochemical cells. Adv. Funct. Mater. **33**(45), 2304067 (2023). <https://doi.org/10.1002/adfm.202304067>
20. Y. Han, J. Zhang, R. Hu, D. Xu, High-thermopower polarized electrolytes enabled by methylcellulose for low-grade heat harvesting. Sci. Adv. **8**(7), eabl5318 (2022). <https://doi.org/10.1126/sciadv.abl5318>
21. H. Wang, X. Zhuang, W. Xie, H. Jin, R. Liu et al., Thermosensitive-CsI_3_-crystal-driven high-power I^−^/I_3_^−^ thermocells. Cell Rep. Phys. Sci. **3**(3), 100737 (2022). <https://doi.org/10.1016/j.xcrp.2022.100737>
22. H. Zhou, T. Yamada, N. Kimizuka, Supramolecular thermo-electrochemical cells: enhanced thermoelectric performance by host–guest complexation and salt-induced crystallization. J. Am. Chem. Soc. **138**(33), 10502–10507 (2016). <https://doi.org/10.1021/jacs.6b04923>
23. A.M. Bates, B. Zickel, S. Krebs, S. Mukherjee, N.D. Schuppert et al., Analytical study and experimental validation of copper II sulfate and potassium ferri/ferrocyanide thermocells using Onsager flux equations. J. Energy Resour. Technol. **139**(4), 042003 (2017). <https://doi.org/10.1115/1.4036045>
24. M. Wu, S. Hao, L. Qi, Y. Shi, W. Yang et al., An N-type thermogalvanic cell with a high temperature coefficient based on the Cu/Cu(en)_22_+ redox couple. ACS Appl. Mater. Interfaces **17**(18), 26775–26783 (2025). <https://doi.org/10.1021/acsami.5c03375>
25. P.F. Salazar, S.T. Stephens, A.H. Kazim, J.M. Pringle, B.A. Cola, Enhanced thermo-electrochemical power using carbon nanotube additives in ionic liquid redox electrolytes. J. Mater. Chem. A **2**(48), 20676–20682 (2014). <https://doi.org/10.1039/c4ta04749d>
26. K. Laws, M.A. Buckingham, M. Farleigh, M. Ma, L. Aldous, High Seebeck coefficient thermogalvanic cells *via* the solvent-sensitive charge additivity of cobalt 1, 8-diaminosarcophagine. Chem. Commun. **59**(16), 2323–2326 (2023). <https://doi.org/10.1039/d2cc05413b>
27. T.J. Abraham, D.R. MacFarlane, J.M. Pringle, High Seebeck coefficient redox ionic liquid electrolytes for thermal energy harvesting. Energy Environ. Sci. **6**(9), 2639–2645 (2013). <https://doi.org/10.1039/c3ee41608a>
28. G.-H. Kim, L. Shao, K. Zhang, K.P. Pipe, Engineered doping of organic semiconductors for enhanced thermoelectric efficiency. Nat. Mater. **12**(8), 719–723 (2013). <https://doi.org/10.1038/nmat3635>
29. Y. Nonoguchi, K. Ohashi, R. Kanazawa, K. Ashiba, K. Hata et al., Systematic conversion of single walled carbon nanotubes into n-type thermoelectric materials by molecular dopants. Sci. Rep. **3**, 3344 (2013). <https://doi.org/10.1038/srep03344>
30. S. LeBlanc, S.K. Yee, M.L. Scullin, C. Dames, K.E. Goodson, Material and manufacturing cost considerations for thermoelectrics. Renew. Sustain. Energy Rev. **32**, 313–327 (2014). <https://doi.org/10.1016/j.rser.2013.12.030>
31. J. Mao, H. Zhu, Z. Ding, Z. Liu, G.A. Gamage et al., High thermoelectric cooling performance of n-type Mg_3_Bi_2_-based materials. Science **365**(6452), 495–498 (2019). <https://doi.org/10.1126/science.aax7792>
32. J. Androulakis, C.-H. Lin, H.-J. Kong, C. Uher, C.-I. Wu et al., Spinodal decomposition and nucleation and growth as a means to bulk nanostructured thermoelectrics: enhanced performance in Pb(1-x)Sn(x)Te-PbS. J. Am. Chem. Soc. **129**(31), 9780–9788 (2007). <https://doi.org/10.1021/ja071875h>
33. L.-D. Zhao, S.-H. Lo, Y. Zhang, H. Sun, G. Tan et al., Ultralow thermal conductivity and high thermoelectric figure of merit in SnSe crystals. Nature **508**(7496), 373–377 (2014). <https://doi.org/10.1038/nature13184>
